# Targeted metabolite profiling as a top-down approach to uncover interspecies diversity and identify key conserved operational features in the Calvin–Benson cycle

**DOI:** 10.1093/jxb/erab291

**Published:** 2021-06-22

**Authors:** Mark Stitt, Gian Luca Borghi, Stéphanie Arrivault

**Affiliations:** 1 Max Planck Institute of Molecular Plant Physiology, Am Mühlenberg 1, D-14476 Potsdam-Golm, Germany; 2 University of Essex, UK

**Keywords:** Calvin–Benson cycle, carbon dioxide, C_3_ and C_4_ photosynthesis, irradiance, metabolite profiling, species diversity

## Abstract

Improving photosynthesis is a promising avenue to increase crop yield. This will be aided by better understanding of natural variance in photosynthesis. Profiling of Calvin–Benson cycle (CBC) metabolites provides a top-down strategy to uncover interspecies diversity in CBC operation. In a study of four C_4_ and five C_3_ species, principal components analysis separated C_4_ species from C_3_ species and also separated different C_4_ species. These separations were driven by metabolites that reflect known species differences in their biochemistry and pathways. Unexpectedly, there was also considerable diversity between the C_3_ species. Falling atmospheric CO_2_ and changing temperature, nitrogen, and water availability have driven evolution of C_4_ photosynthesis in multiple lineages. We propose that analogous selective pressures drove lineage-dependent evolution of the CBC in C_3_ species. Examples of species-dependent variation include differences in the balance between the CBC and the light reactions, and in the balance between regulated steps in the CBC. Metabolite profiles also reveal conserved features including inactivation of enzymes in low irradiance, and maintenance of CBC metabolites at relatively high levels in the absence of net CO_2_ fixation. These features may be important for photosynthetic efficiency in low light, fluctuating irradiance, and when stomata close due to low water availability.

## Teaser

Metabolite profiling has uncovered unexpected diversity of Calvin–Benson cycle operation in C_3_ species, but also conserved features that may be important in the fluctuating conditions found in the field.

## Introduction

Photosynthesis is central to plant growth but uses light energy rather inefficiently, is associated with major water loss, and requires a substantial part of the plant’s nitrogen resources (Long *et al.*, 2006; Zhu *et al.*, 2008; Raines, 2011). Engineering photosynthesis is therefore a promising route to improve crop yield (Long *et al.*, 2015; Ort *et al.*, 2015; Niinemets *et al.*, 2017). Success depends on sound understanding of the physiological, ultrastructural, anatomical, and phenological features that allow effective interception of light and entry of CO_2_, and of the biophysical and biochemical processes that use light energy to convert CO_2_ into carbohydrates and other products. There is considerable diversity in photosynthesis across life forms, species, and even within species (Evans, 1989; Wullschleger, 1993; Wright *et al.*, 2004; Hikosaka *et al.*, 2010; Lawson *et al.*, 2012; Acevedo-Siaca *et al.*, 2020, 2021; McAusland *et al.*, 2020; Silva-Pérez *et al.*, 2020). It is important to gain a deeper and mechanistic understanding of this diversity, as the best engineering strategy may vary from crop to crop and because understanding diversity within a species may open up new avenues for crop breeding.

The Calvin–Benson cycle (CBC) uses NADPH and ATP from the light reactions to drive the incorporation of CO_2_ into phosphorylated intermediates, and is at the core of photosynthesis. This review first summarizes background information about the structure and regulation of the CBC, and on the operation of various carbon-concentrating mechanisms (CCMs) that support CBC operation in our low CO_2_ world. We then survey the evidence for species diversity in photosynthesis, in particular in how the CBC operates. ‘Operation’ refers to the balance between different reactions or the poising of metabolic state in a pathway; species variation can result from differences in the relative abundance of enzymes, in the kinetic and other properties of enzymes, or in the regulatory network that coordinates flux at different sites in and around a pathway. Until now, most of the evidence for CBC diversity came from analyses of whole-leaf photosynthetic traits, and studies of the kinetic and regulatory characteristics of individual CBC enzymes, especially Rubisco. The main part of this review explains how metabolite profiling provides a complementary top-down approach to detect interspecies diversity in CBC operation and to identify features that are conserved across species. This provides a starting point to formulate testable hypotheses about the underlying mechanisms and biological reasons for this combination of diversity and conservation.

## Role and regulation of the Calvin–Benson cycle

**Fig. 1.**
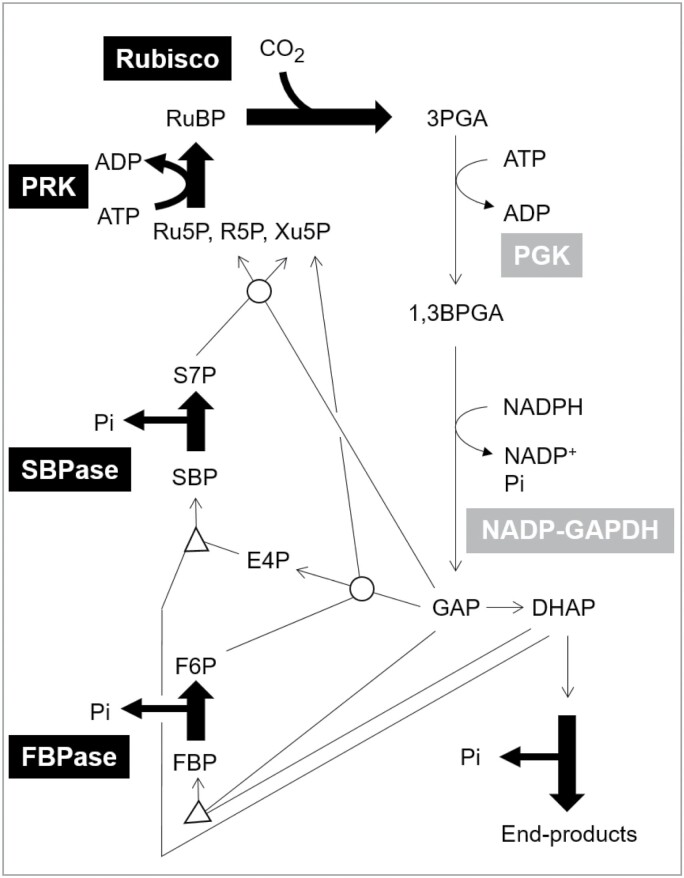
Schematic representation of the Calvin–Benson cycle. This display highlights the location of CBC metabolites and reactions that catalyse irreversible reactions *in vivo* (thick black arrows; name of enzyme in black box). Reactions that catalyse reversible reactions are shown with thin lines, with the arrow indicating the direction of net flux during operation of the CBC. The enzymes involved in 3PGA reduction are named; the reactions catalysed by transketolase and aldolase are identified by a circle and a triangle, respectively. Triose-P isomerase is indicated with an arrow, and the isomerases that interconvert Xu5P, R5P, and Ru5P are omitted for simplicity, as is the stoichiometry of the reactions. In the absence of photorespiration, the CBC catalyses a net reaction of 6NADPH+9ATP+3CO_2_→6NADP^+^+9ADP+8Pi+1triose-P. The remaining Pi is recycled during end-product synthesis, which is indicated here schematically for conversion of triose-P (GAP and DHAP) to sucrose. Some end-product synthesis starts from F6P for starch, 3PGA for organic acids, amino-derived amino acids (E4P) for aromatic acids and phenylpropanoids, and pentose-P for nucleotides. Rubisco also catalyses a reaction with oxygen leading to formation of 2PG+3PGA. The 2PG is scavenged by the photorespiration pathway, in which two 2PG are converted to 3PGA with loss of CO_2_ and NH_3_. When this happens, the flux in the CBC must be increased relative to end-product synthesis to regenerate the RuBP that is consumed in the oxygenase reaction. Abbreviations: 1,3-bisphosphoglyceric acid (1,3BPGA); 3-phosphoglycerate (3PGA); dihydroxyacetone phosphate (DHAP); erythrose 4-phosphate (E4P); fructose 6-phosphate (F6P); fructose-1,6-bisphosphatase (FBPase); fructose 1,6-bisphosphate (FBP); glyceraldehyde 3-phosphate (GAP); glucose 1-phosphate (G1P); inorganic phosphate (Pi); NADP-glyceraldehyde-3-phosphate dehydrogenase (NADP-GAPDH); phosphoglycerate kinase (PGK); phosphoribulokinase (PRK); ribose 5-phosphate (R5P); ribulose 5-phosphate (Ru5P); ribulose 1,5-bisphosphate (RuBP); sedoheptulose 7-phosphate (S7P); sedoheptulose-1,7-bisphosphatase (SBPase); sedoheptulose 1,7-bisphosphate (SBP); xylulose 5-phosphate (Xu5P).

The CBC consists of three subprocesses; fixation of CO_2_ by Rubisco to form two molecules of glycerate 3-phosphate (3PGA), reduction of 3PGA to triose phosphate (triose-P) using NADPH and ATP from the light reactions, and a series of reactions that convert triose-P to ribulose 1,5-bisphosphate (RuBP) (Fig. 1; Edwards and Walker, 1983; Heldt *et al.*, 2005; Stitt *et al.*, 2010; Adam, 2017). Most of the NADPH and about two-thirds of the ATP from the light reactions are used to reduce 3PGA, and most of the remaining ATP is used to convert ribulose 5-phosphate (Ru5P) to RuBP. The net result is the conversion of 6NADPH+9ATP+3CO_2_ into 6NADP^+^+9ADP+8P_i_ (inorganic phosphate), and one triose-P. The remaining Pi is recycled during the conversion of triose-P to end-products. In source leaves of terrestrial plants, the main end-products are carbohydrates such as sucrose and starch, as well as smaller amounts of organic acids and amino acids.

The CBC must be tightly regulated for several reasons (Woodrow and Berry, 1988; Scheibe, 1991; Fridlyand and Scheibe, 1999; Stitt *et al.*, 2010). The first reason is to ensure that sufficient 3PGA is available to utilize the available NADPH and ATP and minimize wastage of energy or damage due to electron pressure in the light reactions. The second is to ensure that RuBP regeneration is fast enough to saturate Rubisco-binding sites. Efficient entry of inorganic carbon is promoted by efficient utilization of CO_2_ by Rubisco; in C_3_ species, this generates the diffusion gradient that drives CO_2_ entry, and in species that operate a CCM it decreases wasteful back-leakage of CO_2_. Rubisco has a low catalytic rate (*k*_cat_) and represents a large part of the total protein in photosynthetic cells (see below for more details). Entry of CO_2_ into the leaf is therefore best promoted by ensuring efficient use of Rubisco rather than having even higher Rubisco abundance. The third reason is to deactivate Rubisco in conditions, such as low irradiance, in which RuBP cannot be regenerated quickly enough to keep all of the Rubisco-binding sites saturated. This is important because there is a high concentration of Rubisco-binding sites. Unless surplus active sites are rendered temporarily inoperative, they will sequester other CBC metabolites and impair pathway flux (see below for more details). The fourth reason is to coordinate fluxes at different reactions around the CBC. A combination of high flux and small metabolite pool sizes means that most CBC metabolites have half-lives of well under 1 s (Stitt *et al.*, 1980, 2010; Arrivault *et al.*, 2009). Flux at different reactions must be tightly coordinated to avoid overaccumulation or depletion of metabolites in different parts of the CBC, which is likely to result in inhibition of pathway flux. This will be especially challenging in fluctuating conditions, for example fluctuating light due to changing cloud cover or the complex light regimes that are found in plant canopies (Vialet-Chabrand *et al.*, 2017; Burgess *et al.*, 2019; Taylor and Long, 2017; Townsend *et al.*, 2018). The fifth reason is to balance the rate at which triose-P and other CBC metabolites are withdrawn for end-product synthesis with the rate of CO_2_ fixation, whilst maintaining the pool sizes of CBC intermediates in a range that supports optimal flux in the CBC (Stitt, 1990; Stitt *et al.*, 2010). The CBC is an autocatalytic cycle, and enough triose-P must be retained in the CBC to regenerate RuBP. It is, however, vital for continued CBC activity that the net gain is exported and converted to end-products, in order to recycle Pi and allow continued synthesis of ATP (Edwards and Walker, 1983; Heldt *et al.*, 2005; McClain and Sharkey, 2019). The sixth reason is to prevent futile cycles and wastage of energy, especially in darkness and, probably, low irradiance. Fluxes in the CBC are ≥50-fold higher than those in respiratory metabolism. Several of the reactions in the CBC potentially form wasteful futile cycles with reactions in respiratory metabolism, and must be strongly inhibited in darkness and low light to avoid large-scale waste of energy and a major loss in energy efficiency (Laing *et al.*, 1981; Heldt *et al.*, 2005).

The CBC contains four essentially irreversible reactions, three of which are involved in the conversion of triose-P to RuBP [plastidic fructose-1,6-bisphosphatase (FBPase), sedoheptulose-1,7-bisphosphatase (SBPase), and phosphoribulokinase (PRK)] and Rubisco (Bassham and Krause, 1969; Dietz and Heber, 1984; Arrivault *et al.*, 2009; Mettler-Altmann *et al*., 2014). 3PGA reduction and the remaining reactions in the regeneration of RuBP are essentially reversible (Fig. 1). Plastidic FBPase, SBPase, and PRK are regulated by multiple mechanisms (Woodrow and Berry, 1988; Fridlyand and Scheibe, 1999; Buchanan and Balmer, 2005; Heldt *et al.*, 2005; Stitt *et al.*, 2010; Michelet *et al.*, 2013; Knuesting and Scheibe, 2018). All three enzymes are activated in the light by rising pH and Mg^2+^ in the stroma, and by thioredoxin-dependent post-translational redox modification. They are subject to feedback regulation by their products or other metabolites. In addition, plastidic FBPase and SBPase have sigmoidal substrate saturation kinetics. These various mechanisms interact closely. For example, in many cases, rising pH and Mg^2+^ alter the concentration of the metabolite form that is the actual substrate or ligand, post-translational activation is often promoted by increased substrate concentration, and post-translational activation often increases substrate affinity. These interactions facilitate a strong increase in flux at plastidic FBPase, SBPase, and PRK as CBC metabolite levels rise. In situations where flux at the three enzymes becomes unbalanced and individual metabolites start to accumulate or be depleted, these interactions facilitate rapid adjustment of the activities of individual enzymes to rebalance flux and metabolite levels around the whole pathway (Stitt *et al.*, 2010; Knuesting and Scheibe, 2018; see below for more details).

Rubisco is in some ways a special case. Due to its relatively low *k*_cat_ (see below for more discussion), Rubisco is present at much higher concentrations than other CBC enzymes. A recent quantitative proteomics analysis in the green alga *Chlamydomonas reinhardtii* (Hammel *et al.*, 2020) found that Rubisco represented 8.4% of total protein, 68% of the total protein in CBC enzymes, and was 6- to 280-fold higher than any other individual CBC enzyme. The dominance of Rubisco is even larger in terrestrial C_3_ species where it represents ≥20% of total leaf protein (Ellis, 1979; Evans, 1989; see also below). It is important to maintain RuBP at levels that saturate Rubisco-binding sites because other CBC intermediates can bind to Rubisco (Badger and Lorimer, 1981). Rubisco is also prone to side reactions including formation of a tight binding inhibitor, pentadiulose 1,5-bisphosphate, that, on average, is formed once in every 260 turnovers (Kane *et al.*, 1998; Pearce and Andrews, 2003) and whose removal requires Rubisco activase, a dedicated molecular chaperone. Rubisco activase is itself regulated by thioredoxin and the ATP/ADP ratio (Zhang and Portis, 1999; Portis and Parry, 2007; Portis *et al.*, 2008). This allows deactivation of Rubisco when its activity exceeds that required in the prevailing environment (Sage *et al.*, 1990; Sage and Seeman, 1993; Parry *et al.*, 2008). In many, but not all, species, Rubisco is also inhibited by sugar phosphates that are synthesized and degraded in a light-dependent manner, such as d-xylulose-1,5-bisphosphate and 2-carboxy-d-arabinitol 1-phosphate (Moore *et al.*, 1993, 1995; Andralojc *et al.*, 1996, 2002; Charlet *et al.*, 1997; Parry *et al.*, 2008).

The model of Farquhar and colleagues (Farquhar *et al.*, 1980; von Caemmerer and Farquhar, 1981) provides a powerful context for understanding the regulation and operation of the CBC. The model links pathway structure and key elements of enzyme kinetics, especially those of Rubisco, with the observed response of photosynthesis to irradiance and CO_2_ supply. It identifies two basic states of the CBC; one in which photosynthesis is limited by Rubisco, for example in saturating light or low CO_2_, and one in which photosynthesis is limited by the rate of RuBP regeneration, for example in limiting light (Badger *et al.*, 1984; Sharkey, 1985). The central role played by Rubisco in this model is fully consistent with its low *k*_cat_ and high abundance (see above), and its kinetic characteristics (see below). In some conditions, photosynthesis may also be limited by the rate of end-product synthesis and the associated recycling of Pi. This is termed triose-P utilization limitation of photosynthesis. It is seen most clearly after transients that increase the rate of photosynthesis, with a balance often being re-established in the mid-term in which the rate of end-product synthesis somewhat exceeds the rate of photosynthesis (Sharkey, 1985, 2019; MacClain and Sharkey, 2019).

Notwithstanding the crucial role of Rubisco, photosynthesis depends on efficient conversion of triose-P to RuBP. Efficient removal of triose-P will promote 3PGA reduction and the associated consumption of NADPH and ATP from the light reactions, and efficient formation of RuBP is essential to saturate Rubisco-binding sites and promote entry of CO_2_ (see above). These considerations were formulated by Woodrow and Berry (1988) and received experimental support in the following 20 years from several groups that investigated the flux control coefficients (FCCs) of CBC enzymes (summarized in Stitt and Schulze, 1994; Stitt and Sonnewald, 1995; Stitt *et al.*, 2010; Raines, 2011). Briefly, these studies created sets of transgenic plants with a progressive decrease in the abundance of a given CBC enzyme, and analysed the impact on the rate of photosynthesis. Such analyses provide several insights into regulation. First, they reveal whether a given enzyme restricts the rate of photosynthesis or if it is in excess. In the latter case, they also reveal that much of the enzyme can be removed before it starts to restrict photosynthesis. Second, for an enzyme that is restricting the rate of photosynthesis, they reveal whether the enzyme is strictly limiting, in which case a small decrease in abundance leads to a proportionally similar decrease in the rate of photosynthesis (technically, FCC=1 or close to it) or, if the enzyme is co-limiting, in which case a small decrease in its abundance leads to a perceptible but smaller decrease in the rate of photosynthesis (technically FCC is clearly less than 1 but above 0).

Analysis of the FCCs of CBC enzymes confirmed an important role for Rubisco, but also underlined that Rubisco is seldom fully limiting for photosynthesis (Quick *et al.*, 1991; Mate *et al.*, 1993; Stitt and Schulze, 1994). Many other CBC enzymes can be co-limiting or are not present in large excess; examples included aldolase (Haake *et al.*, 1998, 1999), transketolase (Henkes *et al.*, 2001), plastidic FBPase (Kossmann *et al.*, 1994), and in particular SBPase (see Harrison *et al.*, 1998; Miyagawa *et al.*, 2001; Lefebvre *et al.*, 2005; Driever *et al.*, 2017; Simkin *et al.*, 2017; also in algae, see Hammel *et al.*, 2020). A co-limiting role for SBPase was also predicted by evolutionary modelling (Zhu *et al.*, 2007). Overall, these studies pointed to an important role for several CBC enzymes and especially SBPase in co-limiting flux in the CBC.

These analyses also revealed that the distribution of control (i.e. the FCC values of different enzymes) depends strongly on both current conditions and past history (see, for example, Stitt and Schulze, 1994). For example, suddenly transferring tobacco from low light growth conditions to high light led to an increase of the FCC of Rubisco from near zero to >0.8 (Stitt and Schulze, 1994) and of the FCC of PRK from zero to 0.23 (Paul *et al.*, 2000). On the other hand, when plants were grown in high light, the FCC of Rubisco was ~0.5. This implies that the optimal balance between the abundance of the various CBC enzymes depends on the conditions. Under fluctuating conditions in the field and especially in canopies (see Pearcy, 1990; Zhu *et al.*, 2004; Slattery *et al.*, 2018; De Souza *et al.*, 2020), an optimal balance may not be achieved across all conditions. This is because CBC enzymes are not subject to rapid turnover (Gibon *et al.*, 2004; Baerenfaller *et al.*, 2015; Li *et al.*, 2017), precluding rapid adjustment of relative abundances when conditions change. A theoretical analysis of alternating high and low light at different frequencies and its implication for the optimal balance between abundance of Rubisco and Rubisco activase can be found in Mott and Woodrow (2000).

Given the mounting interest in understanding photosynthesis in field conditions (Kromdijk *et al.*, 2016; Vialet-Chabrand *et al.*, 2017; Taylor and Long, 2017; Townsend *et al.*, 2018; Burgess *et al.*, 2019), it would be instructive to investigate more systematically the impact of decreased abundance of CBC enzymes on aggregated photosynthetic performance and growth in fluctuating regimes such as those experienced in the field.

## The side reaction of Rubisco with oxygen and its consequences for CBC operation and the evolution of photosynthesis

Rubisco catalyses a side reaction with O_2_, leading to formation of 2-phosphoglycolate (2PG) (Bowes and Ogren, 1972; Andrews *et al.*, 1973; Lorimer, 1981; Tcherkez, 2015) that must be salvaged via the wasteful photorespiratory pathway, in which two 2PG molecules are recycled to one 3PGA molecule with concomitant loss of CO_2_ and ammonia (Bauwe, 2018; Busch, 2020). The oxygenase reaction would have been suppressed in the high-CO_2_ low-O_2_ atmosphere which prevailed 2.7 million years ago when the CBC evolved in photosynthetic bacteria (Rasmussen *et al.*, 2008). However, oxygenic photosynthesis led to a massive decrease in the atmospheric CO_2_ and increase in the atmospheric O_2_ concentration. In current atmospheric conditions (~400 ppm CO_2_, 21% O_2_), about every fourth reaction of Rubisco is with O_2_ (Osmond, 1981; Sharkey, 1988; Hagemann *et al.*, 2016; Betti *et al.*, 2016).

Over the last 2 billion years, photosynthesis has been subject to continued and massive selection due to falling CO_2_ and rising O_2_ concentration in the atmosphere, as well as changes in temperature and the water and nutrient supply (Raven *et al.*, 2017; Sage, 2017). Rubisco had a rather low rate of catalysis with a median *k*_cat_ value of ~3.3 s^−1^, which is about three times slower than the median of enzymes (Bar-Even *et al.*, 2011; Davidi *et al.*, 2018). Against this background, during evolution there have been large changes in the structure and characteristics of Rubisco, with the higher plant Rubisco having a higher selectively for CO_2_ over O_2_, but a lower *k*_cat_ than that of ancestral algae (Jordan and Ogren, 1981; Badger *et al.*, 1998; Savir *et al.*, 2010; Shih *et al.*, 2016; Sharwood *et al*., 2016a, b; Erb and Zarzycki, 2018; Iniguez *et al*., 2020). Higher selectively for CO_2_ is thought to be mechanistically linked to a decrease in *k*_cat_ (Tcherkez *et al.*, 2006; Savir *et al.*, 2010; Bathellier *et al.*, 2018; but see also Flamholz *et al.*, 2019). As Rubisco is the most abundant protein in plant leaves (Ellis, 1979) and across the globe (Bar-On and Milo, 2019), this trade-off between selectivity and *k*_cat_ impacts the nitrogen use efficiency of photosynthesis at the level of the plant, ecosystem, and globe.

Several globally important groups of photosynthetic organisms have evolved CCMs that concentrate CO_2_ and partly suppress the wasteful side reaction with O_2_. Algal CCMs probably evolved in response to the equimolar concentrations of CO_2_ and O_2_ in surface waters ~500 million years ago (Griffiths *et al.*, 2017). Cyanobacteria actively concentrate bicarbonate into carboxysomes, where it is converted to CO_2_ by carbonic anhydrase (CA) to generate a high concentration of CO_2_ around Rubisco (Badger *et al.*, 1998; Kerfeld and Melnicki, 2016). Many green eukaryotic algae accumulate bicarbonate in pyrenoids, where CA again generates a high concentration of CO_2_ around Rubisco (Badger *et al.*, 1998; Wang *et al.*, 2015; Meyer *et al.*, 2017; Griffiths *et al.*, 2017). Another CCM, termed C_4_ photosynthesis, utilizes a biochemical pump to concentrate CO_2_ around Rubisco. C_4_ photosynthesis evolved in some terrestrial angiosperms 25–30 million years ago in response to a transition in the Earth’s climate from hot and wet conditions with atmospheric CO_2_ concentration of >1000 ppm to cooler and drier conditions and CO_2_ concentrations <300 ppm (Christin *et al.*, 2008; Zachos *et al.*, 2008). In C_4_ plants, Rubisco and the remainder of the CBC are restricted to enlarged bundle sheath cells located around the vasculature in the centre of the leaf. The CCM starts with assimilation of bicarbonate by phospho*enol*pyruvate (PEP) carboxylase in the mesophyll to generate 4-carbon metabolites that diffuse to the bundle sheath where they are decarboxylated to release CO_2_ and 3-carbon metabolites, which move back to the mesophyll (Hatch, 2002; von Caemmerer and Furbank, 2003; Sage *et al.*, 2012; Sage, 2017; Arrivault *et al.*, 2019; Schlüter and Weber, 2020). C_4_ photosynthesis is a complex trait whose evolution required several pre-conditioning steps including closer spacing of leaf veins, the establishment of a proto-Kranz anatomy with large or plentiful bundle sheath cells that have evolved from an essentially non-photosynthetic cell into a photosynthetic cell, acquisition of genetic regulatory elements, and extensive genomic duplication (Langdale, 2011; Sage *et al.*, 2012). It evolved via a stepwise evolutionary process that combined flexibility in the order of recruitment of different subtraits (Williams *et al.*, 2013) with a progressive gain in fitness as successive traits were added (Mallmann *et al.*, 2014). C_4_ photosynthesis evolved independently in 65 separate lineages, representing ~3% of terrestrial plant species (Sage *et al.*, 2011; Sage, 2017). Even after establishment in a lineage, C_4_ photosynthesis continued to evolve and diversify (Bianconi *et al.*, 2020). Depending on the lineage, different combinations of anatomy and pathways are deployed to achieve a common goal of concentrating CO_2_ in the bundle sheath cells (Furbank, 2011; Sage *et al.*, 2011; Sage, 2017). For example, depending on the species, the decarboxylation reaction can occur via NADP-malic enzyme (NADP-ME), NAD-malic enzyme (NAD-ME), PEP carboxykinase (PEPCK), or a combination of these (Furbank, 2011; Bräutigam *et al.*, 2014, 2018, Preprint) with accompanying differences in which metabolites move between the mesophyll cell and bundle sheath cells, and whether there are modifications of the light reactions in the bundle sheath cells.

CCMs will impact CBC operation. One well-characterized impact is that under high CO_2_ concentrations Rubisco can evolve back to a lower selectivity and higher *k*_cat_ form, with an associated decrease in the amount of nitrogen that must be invested in Rubisco to achieve a given rate of photosynthesis. This has been well documented both in green algae with pyrenoids (Meyer and Griffiths, 2013; Heureux *et al.*, 2017; Goudet *et al.*, 2020) and in terrestrial C_4_ plants (Brown, 1978; Yeoh *et al.*, 1980; Sage and Seemann, 1993; Badger *et al.*, 1998; Carmo-Silva *et al.*, 2010; Kapralov *et al.*, 2011; Sharwood *et al.*, 2016). Little is known about whether CCMs require or permit further modifications of the CBC. One well-characterized case is some NADP-ME subtypes of the C_4_ syndrome, where loss of PSII activity in the bundle sheath chloroplasts requires that about half of the 3PGA that is produced by Rubisco must move to the mesophyll chloroplasts, where it is reduced to triose-P that moves back to the bundle sheath cells (Hatch, 2002; von Caemmerer and Furbank, 2003; Bräutigam *et al.*, 2018, Preprint). This shuttle requires close coordination of metabolism in two different cell types, including the generation of concentration gradients of 3PGA from the bundle sheath to the mesophyll cells, and of triose-P from the mesophyll to the bundle sheath cells (Leegood, 1985*a*, *b*; Stitt and Heldt, 1985; Arrivault *et al.*, 2017). It presumably depends on efficient reduction of 3PGA in the mesophyll chloroplasts, and efficient utilization of triose-P in the bundle sheath chloroplasts.

## Diversity of CBC operation between C_3_ species

The vast majority of terrestrial C_3_ species lack a CCM but will also have been subject to selective pressure by falling CO_2_, as well as changing temperature and changing water and nutrient availability. Little is known about whether these evolutionary pressures drove changes in the CBC. However, this appears likely, for example, because of the importance of optimizing RuBP regeneration to allow efficient operation of Rubisco (see above). There is considerable diversity in the rate of photosynthesis between terrestrial C_3_ species including closely related species and even cultivars or accessions from the same species (Evans, 1989; Wullschleger, 1993; Gu *et al.*, 2012; Driever *et al.* 2014; Sakoda *et al.*, 2016; Acevedo-Siaca *et al.*, 2020, 2021; McAusland *et al.*, 2020; Silva-Pérez *et al.*, 2020). Several contributing factors have been identified, including differences in the rate of electron transport and carboxylation (Wullschleger, 1993), differing leaf nitrogen content, allocation, and use efficiency (Field and Mooney, 1986; Evans, 1989; Hikosaka, 2010), and differing investment strategies between short-lived and long-lived leaves (Wright *et al.*, 2004; Donovan *et al.*, 2011). In rice and wheat, photosynthesis traits even vary independently in the flag leaf and the leaf located immediately below the flag leaf (Acevedo-Siaca *et al.*, 2020, 2021). This observation points to an interaction between genotype and either leaf development or local differences in the environment experienced by the two leaf types.

These interspecies differences in the rate of photosynthesis will require changes in CBC flux. Some might even be partly due to changes in the capacity, properties, or regulation of the CBC. For example, there is marked diversity in the kinetic characteristics of Rubisco between C_3_ species (Galméz *et al*., 2014a, b; Carmo-Silva *et al*., 2015; Orr *et al.*, 2016; Prins *et al.*, 2016; Hermida-Carrera *et al.*, 2017) and the properties of Rubisco activase (Carmo-Silva and Salvucci, 2013). In some cases, species differences in the kinetic properties of Rubisco could be linked back to changes in amino acid sequence, both in C_3_ plants (Orr *et al.*, 2016) and at a broader scale across all photosynthetic life forms (Davidi *et al.*, 2020). There are also striking differences between C_3_ species regarding which Rubisco-inhibitory sugar bisphosphates they contain (Servaites *et al.*, 1986; Moore *et al.*, 1993; Parry *et al.*, 2008; Carma-Silva and Salvucci, 2013) of Rubisco. As a further example, the function of the regulatory CP12 protein differs between species (Howard *et al*., 2011; Gontero and Maberly, 2012; López-Calcagno *et al*., 2014).

## Metabolite profiling as a top-down strategy to uncover interspecies variance in CBC operation

The research discussed in the preceding sections documents that there is diversity in CBC operation, both between different C_3_ species, and between C_3_ species and species that operate a CCM. However, at a mechanistic level, most previous work focused on individual enzymes, especially Rubisco. There is a need for unbiased top-down experimental approaches that screen for changes in CBC operation and provide information about which enzymes may be involved.

For >60 years, systematic measurements of metabolite levels—now termed metabolite profiling—have been used to identify enzymes that are regulated when fluxes respond to a change in the environment or a developmental transition (Rolleston, 1972; Newsholme and Start, 1973). The principle is analogous to observing the density of automobiles in a network of roads in order to identify congestion sites and inform drivers to take another route. In a scenario where pathway flux is decreased, enzymes that have been inhibited can be identified because their substrates rise and their products remain unaltered or fall. In a scenario where pathway flux is increased, enzymes that have been activated can be identified because their substrates fall, and their products stays unaltered or rise. This approach is more suitable for enzymes that catalyse irreversible reactions than for enzymes that catalyse reactions which are close to thermodynamic equilibrium, because in the latter case the substrate and product tend to change in parallel with each other. Importantly, this approach identifies which enzymes are being regulated, irrespective of what kind of regulatory mechanism is involved. It short-lists enzymes that are being regulated, and can be followed by mechanistic studies of the enzymes to discover the regulatory mechanism(s). Early examples of the application of this approach in photosynthesis led to the discovery that plastidic FBPase and SBPase are regulated by the stromal pH and Mg^2+^ concentration (Werdan *et al.*, 1975; Portis and Heldt, 1976).

Metabolite profiling also provides an unbiased and top-down strategy to search for interspecies variance in how a pathway is operating. Changes in the levels of pathway intermediates between different species will highlight changes in the balance between different enzymatic steps. Importantly, metabolite profiling detects variation, irrespective of whether the variation is due to changes in gene expression and protein abundance, enzyme kinetics, or regulatory properties, or to changes in the structure of the network that regulates the pathway and coordinates it with other processes.

## Measuring CBC metabolites is challenging

This top-down approach has not yet been used in a systematic manner to compare CBC operation in different species. One reason is that collecting reliable quantitative data on all, or almost all, of the CBC intermediates is not trivial. The earliest comprehensive analyses of CBC intermediates were obtained by Bassham and colleagues in the 1960s, using ^14^CO_2_ labelling of *Chlorella pyrenoidosa* followed by paper chromatography and autoradiography (Bassham and Kirk, 1960; Bassham and Krause, 1969). These studies built on the analytic technology that was used to discover the CBC (Benson, 2002) but, instead of using of short pulses, cells were labelled with ^14^CO_2_ for long enough to reach steady-state isotopic labelling. These methods were technically challenging and have not been applied to a range of species, let alone to higher plants. In the 1970s, Heldt and colleagues used ^32^P labelling followed by LC coupled to a flow-through detector to profile CBC metabolites (Lilley *et al.*, 1977). This method was applied to study CBC regulation in isolated chloroplasts but was not applicable in intact cells or organisms because it is hardly possible to safely label them to steady state with ^32^P. Use of analogous approaches with ^14^CO_2_ were precluded by rapid labelling of many metabolites outside the CBC, which interfered with reliable detection of CBC metabolites based on chromatographic mobility alone. For this reason, studies of CBC metabolite levels in leaves in the last decades of the 20th century used enzymatic metabolite assays (Stitt *et al.*, 1980, 1983, 1984; Badger *et al.*, 1984; Dietz and Heber, 1984; von Caemmerer and Edmondson, 1986; Seeman and Sharkey, 1986, 1987; Sharkey and Seeman, 1989; Servaites *et al.*, 1989; compiled in Borghi *et al.*, 2019). Enzymatic assays were only available for some metabolites, so these analyses were limited to RuBP and a handful of other intermediates [3PGA, triose-P, FBP, and fructose 6-phosphate (F6P), whereby FBP was not fully separated from SBP]. In the last 15 years, new analytical platforms have been developed that combine chromatographic separation with tandem mass spectrometry (LC-MS/MS) allowing near-comprehensive analysis of CBC intermediates (Cruz *et al.*, 2008; Arrivault *et al.*, 2009; Hasunuma *et al.*, 2010; Ma *et al.*, 2014; Xu *et al.*, 2021). Importantly, when combined with the use of isotopically labelled standards, these platforms provide quantitative data that can be compared across different species with differing background composition and potential issues with ion suppression (Arrivault *et al.*, 2015).

Another challenge in applying metabolite profiling to the CBC is posed by the very short half-time of CBC intermediates (<1 s and many closer to 0.1 s; see above). This means that even temporary changes in irradiance or CO_2_ levels during the quenching process will lead to changes in metabolite levels. Various systems have been developed to circumvent this problem, including spraying algal suspension under ambient irradiance into very cold methanol (Bassham and Krause, 1969; Mettler-Altmann *et al*., 2014) and, for leaves, instantaneous freezing between two metal columns that have been pre-cooled to liquid N_2_ temperature (Badger *et al.*, 1984) and flooding of small containers with liquid N_2_ (Arrivault *et al.*, 2009, 2017; Szecowka *et al.*, 2013; Ermakova *et al.*, 2021; Xu *et al.*, 2021).

## First application of CBC metabolite profiling to terrestrial C_3_ and C_4_ species

Arrivault *et al.* (2019) compared the profiles of CBC intermediates in four C_4_ species [*Zea mays* (maize), *Setaria viridis*, *Flaveria bidentis*, and *F. trinervia*, all in the NADP-ME subtype] and five C_3_ species [*Oryza sativa* (rice), *Triticium aestivum* (wheat), *Arabidopsis thaliana* (Arabidopsis), *Nicotiana tabacum* (tobacco), and *Manihot esculenta* (cassava)]. This set of species included monocot and dicot species for each photosynthesis type, several model species for photosynthesis research (maize, Arabidopsis, and tobacco) and several important crops (maize, rice, wheat, and cassava). The analyses focused on CBC intermediates, in order to avoid distortion by species differences in ancillary or unrelated pathways.

Cross-species comparisons face potential pitfalls. One is the choice of the growth and harvest conditions. Optimal growth conditions and response of photosynthesis to light and temperature vary between species. Arrivault *et al.* (2019) took a pragmatic approach, growing each species in conditions that suited it and harvesting in growth conditions. Irradiance levels were chosen that were moderately limiting, in which conditions RuBP regeneration would be limiting for photosynthesis (see above). A second potential complication is that leaf composition varies from species to species; for example, in this set of species, protein and chlorophyll were higher on a leaf mass basis in rice and cassava than in the other C_3_ species. This could lead to apparent differences in metabolite profiles that are driven by changes in leaf composition rather than the balance of metabolism in the CBC. To detect and avoid such secondary effects, metabolites were normalized on different parameters; not only fresh weight but also chlorophyll and protein content. They were also normalized by expressing the carbon present in a given metabolite as a fraction of the total carbon in all CBC metabolites. The latter was termed the ‘dimensionless’ normalization and provides information on the distribution of carbon between the CBC metabolites.

**Fig. 2.**
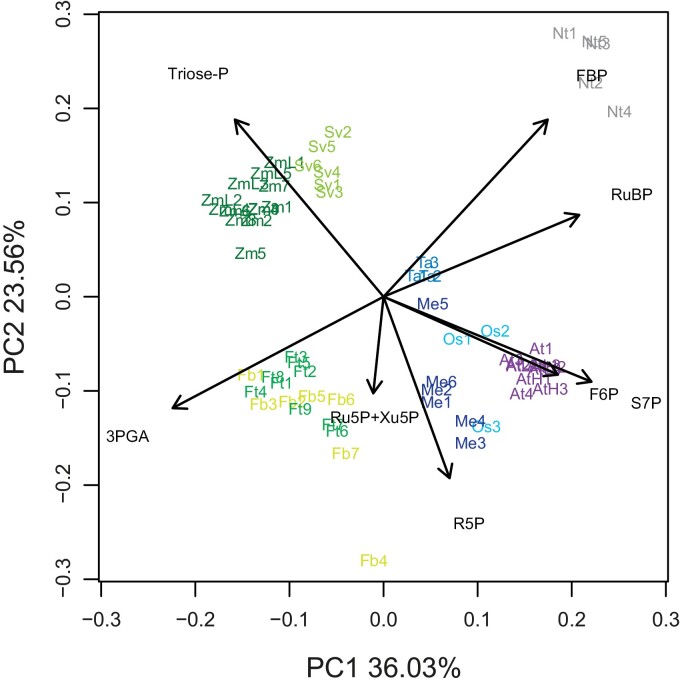
Principal component analysis of CBC metabolite profiles in four C_4_ and five C_3_ species. Metabolite amounts were transformed by multiplying the amount of a given metabolite (nmol g FW^−1^) by the number of carbon atoms in the metabolite to estimate the carbon equivalent content (nmol C g FW^−1^), and then dividing this by the total carbon equivalents in all CBC intermediates plus 2PG. This transformation generates a dimensionless dataset in which each metabolite receives a value equal to its fractional contribution to all the carbon in CBC metabolites plus 2PG. This removes potential bias due to differences in leaf composition. Note that SBP was excluded from the PC analysis shown here because some species appear to contain a subpool of SBP that is not involved in CBC flux (see Arrivault et al., 2017, 2019). The result was, however, not changed greatly when SBP was included (not shown). The distribution of C_4_ species (shades of green) and C_3_ species (shades of blue and grey) is shown on PC1 and PC2 (*Z. mays/*maize, Zm and ZmL; *S. viridis*, Sv; *F. bidentis*, Fb; *F. trinervia*, Ft; *O. sativa*/rice, Os; *T. aestivum*/wheat, Ta; *A. thaliana*/Arabidopsis, AtL, AtM, and AtH; *N. tabacum*/tobacco, Nt; *M. esculenta*/cassava, Me). Each species occupies its own space, except for the two *Flaveria* species which overlap. For Arabidopsis, L, M, and H refer to samples harvested after 15 min in low, medium (=growth), and high light (80, 120, and 280 µmol m^−2^ s^−1^, respectively). For maize, ZmL and Zm refer to samples harvested after 4 h at low irradiance or left at growth irradiance, respectively (160 µmol m^−2^ s^−1^ and 550 µmol m^−2^ s^−1^). Samples group independently of the harvest irradiance. The weightings of CBC intermediates in PC1 and PC2 are shown in black. The display is modified from Arrivault *et al.* (2019). Metabolite abbreviations are as in Fig. 1.

When these cross-species datasets were subjected to principal component (PC) analysis (Fig. 2; this is for the ‘dimensionless’ normalization, see Arrivault *et al.* (2019) for PC analyses with other normalizations), four general conclusions emerged. First, samples for a given species grouped together, showing that within-species noise is smaller than between-species diversity. This is a pre-condition for the approach, and already hints at there being interspecies variation in CBC operation. Second, the four C_4_ species were separated from the five C_3_ species. This is expected as the CBC adjusts to the high CO_2_ environment provided by the C_4_ CCM (see above). Inspection of the metabolite vectors that drive the separation of C_4_ from C_3_ species (Fig. 2) revealed that RuBP is lower in C_4_ species than in C_3_ species; this is expected and presumably reflects the lower abundance of Rubisco (and hence the concentration of RuBP-binding sites) in C_4_ species than in C_3_ species due to the higher *k*_cat_ of C_4_ Rubisco (see above). Third, within the C_4_ species, *Z. mays* and *S. viridis* were separated from the *Flaveria* spp. This was driven by higher 3PGA and triose-P in maize and *S. viridis*. As already mentioned, all four C_4_ species belong to the NADP-ME subtype. Decarboxylation of malate by NADP-ME in the plastids of bundle sheath cells delivers not only CO_2_ but also about half of the NADPH that is required by the CBC. Depending on the species, this has allowed partial or complete loss of PSII in the bundle sheath chloroplasts. Maize has bimorphic chloroplasts lacking PSII in the bundle sheath. The other half of the NADPH that is required by the CBC is imported from the mesophyll cells via an intercellular 3PGA/triose-P shuttle. This requires elevated overall contents of these metabolites to generate the necessary intercellular concentration gradients (see above). The high 3PGA and triose-P in *S. viridis* indicate that a similar shuttle operates in this species. The two C_4_*Flaveria* species have PSII activity in their bundle sheath chloroplasts to a varying extent depending on conditions (Laetsch and Price, 1969; Höfer *et al.*, 1992; Meister *et al.*, 1996; Nakamura *et al.*, 2013) and probably only operate a partial intercellular 3PGA/DHAP (dihydroxyacetone phosphate) shuttle (Leegood and von Caemmer, 1994). The separation of the four C_4_ species based on known differences in their biochemistry, and the separation of C_4_ species from C_3_ species shows that this rather simplistic analysis can detect interspecies differences in CBC operation. The fourth and maybe more unexpected finding was that the five C_3_ species separated from each other. This was driven by several metabolites including FBP, hexose-P, and pentose-P.

**Fig. 3.**
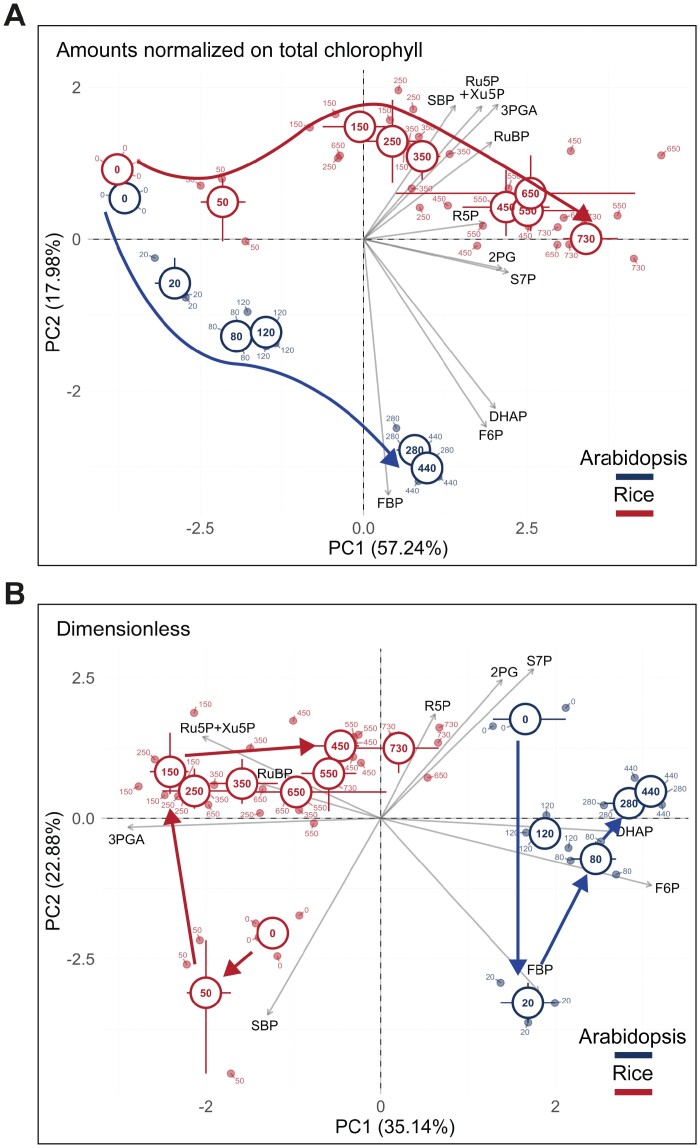
Principal component (PC) analysis of the response of Arabidopsis and rice to rising irradiance. Samples were collected in darkness and at 20, 80, 120, 280, and 420 µmol m^−2^ s^−1^ irradiance in Arabidopsis (shown in blue) and in darkness and at 50, 150, 250, 350, 450, 550, 650, and 730 µmol m^−2^ s^−1^ in rice (shown in red). The lowest irradiance intensity was close to the light compensation point and the others were ~67, 100, 230 and 360% in Arabidopsis and ~30, 60, 80, 100, 120, 150, and 170% in rice of that required to half-saturate photosynthesis (not shown). Positions of individual samples at each irradiance are indicated by the corresponding number in small font. The plots show PC1 and PC2 for analyses performed on (A) chlorophyll-normalized data and (B) a dimensionless dataset (see legend to Fig. 2). The average of these samples in the space defined by PC1 and PC2 is indicated by a circle (irradiance in large font) and the 95% confidence limits are depicted by bars. Metabolite loadings are depicted as grey arrows, and the respective metabolite is shown in black font. The thick red and blue lines show the response of rice and Arabidopsis to increasing irradiance. The unusual asymmetric distribution of loadings in PC1 in the PC analysis with chlorophyll-normalized data reflects a general increase in the levels of most metabolites as irradiance increases. Species-specific responses are captured in PC2 where rice and Arabidopsis take different trajectories as irradiance increases. The masking effect of the general rise in metabolite levels is removed when metabolites are normalized on total carbon content in CBC metabolites at that light intensify (B). This plot reveals that CBC metabolites occupy a different metabolic space in rice and Arabidopsis that is largely independent of irradiance. Note also that the ‘outlier’ at low irradiance at around the light compensation point that is captured in PC2 and is driven by high FBP and high SBP contributes to the separation in PC1. The display is modified from Borghi *et al.* (2019). Metabolite abbreviations are as in Fig. 1.

As already mentioned, cross-species comparisons of metabolite profiles will be problematic if interspecies differences are smaller than the impact of environmental conditions on metabolite levels. Arrivault *et al.* (2019) noted that changes in light intensity did not have a major impact on the CBC intermediate profile. Borghi *et al.* (2019) investigated the impact of irradiance in more detail, transferring Arabidopsis or rice plants to different irradiance for 15 min or 20 min before harvest, respectively. They used light intensities that ranged from the light compensation point up to intensities that allowed near-maximal rates of photosynthesis. Figure 3 shows PC analyses using chlorophyll-normalized and ‘dimensionless’ data. The analysis with chlorophyll-normalized data (Fig. 3A) revealed a general increase in metabolite levels in both species that was captured mainly in PC1 and species-dependent differences that were captured in PC2. The PC analysis with the ‘dimensionless’ dataset (Fig. 3B) removes the effect of the irradiance-dependent increase in metabolites (because each metabolite is normalized on the sum of all metabolites at a given irradiance) and reveals more clearly the species-dependent differences. Arabidopsis and rice occupied a rather similar position in darkness and low light, but took a completely different trajectory with rising irradiance. This separation was mainly captured in PC1 and was driven by several metabolites (see below for further discussion). It reveals that these two species use different strategies to increase CBC flux as more energy becomes available from the light reactions. The PC analysis with the ‘dimensionless’ dataset also revealed a shared response in very low irradiance that was orthogonal to the response at higher irradiance, was captured in PC2, and was driven by high FBP and SBP (see later for further discussion).

## Features of the CBC metabolite profile that vary between C_3_ species

First information about which metabolites are driving the separation of species is provided by inspecting the weighting of PC vectors, and this can be followed up by correlation analysis and detailed visual comparisons of the responses of selected metabolites. Such analyses are aided by background information about the thermodynamic topology of the CBC. Biochemical pathways contain a mix of reversible reactions that are close to thermodynamic equilibrium and whose reactants typically change in parallel with each other, and irreversible reactions that are removed from thermodynamic equilibrium and whose substrates and products can vary independently of each other. As already mentioned, in the CBC there are four irreversible reactions catalysed by plastidic FBPase, SBPase, PRK, and Rubisco. When the dataset of Arrivault *et al.* (2019) was used to identify which pairs or sets of metabolites are positively correlated across species and which are uncorrelated or even show negative correlations, an important general picture emerged. Metabolites that are interconverted by reversible reactions tended to be positively correlated across species. This is to be expected as thermodynamic constraints will apply to all species; indeed, this result provides support for the idea that cross-species comparisons of metabolite profiles will provide reliable information about CBC operation. Metabolite pairs that are linked by irreversible reactions were often correlated poorly or were even negatively correlated across species. This result points to interspecies variation in the balance between plastidic FBPase, SBPase, PRK, and Rubisco. Quite similar results were found when the correlation analysis was performed with a dataset including all species, only C_4_ species, or only C_3_ species (Arrivault *et al.*, 2019). As plants were harvested in steady-state conditions, the relative flux at each enzyme is defined by CBC pathway topology and will be the same for every species. However, depending on the species, different levels of substrates and effector ligands are required to achieve this balance.

**Fig. 4.**
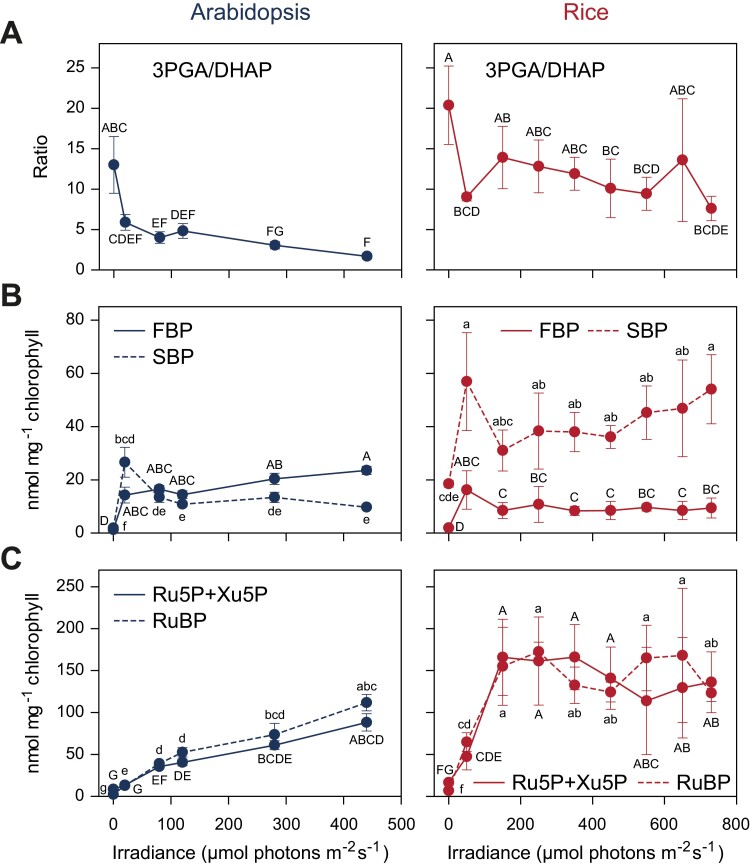
Examples of divergent responses of CBC metabolite levels in rice (blue, left-hand panels) and Arabidopsis (red, right-hand panels). (A) The 3PGA/DHAP ratio. Arabidopsis maintains a lower 3PGA/DHAP ratio over a wide range of irradiance. This points to the balance between the light reactions and the CBC being shifted in favour of the CBC in Arabidopsis and the light reactions in rice. (B) FBP and SBP levels. Arabidopsis has higher levels of FBP than of SBP, and rice has higher levels of SBP than of FBP. These differences are maintained over a wide range of irradiance. This points to the balance between FBPase and SBPase activity being shifted in favour of SBPase activity in Arabidopsis and FBPase activity in rice. (C) Levels of pentose-P and RuBP. These rise with irradiance in Arabidopsis, but plateau at a relatively low irradiance in rice. This points to the rate of CO_2_ fixation by Rubisco being increased, as irradiance rises, in Arabidopsis by increasing levels of RuBP, and in rice by increased activity of Rubisco brought about by means other than substrate availability. The results are shown as the mean ±SD (*n*=4 in almost all cases). One-way ANOVA with false disovery rate (FDR) was performed for each metabolite on the entire dataset after log transformation, treating each species–irradiance combination as a separate treatment. This tests whether responses of a given species to changes in irradiance are significant, and if metabolite levels differ between species, whereby the latter comparison can be made independently of absolute irradiance. This was followed by a Tukey’s HSD post-hoc test. Treatments that are not significantly different share a letter. As two different metabolites are shown in each panel, the Tukey’s HSD results are shown with different cases for the two metabolites (e.g. ‘a’ and ‘A’). Letters are assigned such that ‘a’ or ‘A’ denote the treatment group with the highest level. The display is modified from Borghi *et al.* (2019). Metabolite abbreviations are as in Fig. 1.

The comparison of the response of Arabidopsis and rice to rising irradiance in Borghi *et al.* (2019) provided more detailed insights into how CBC operation differs between these two C_3_ species. Three examples are shown in Fig. 4. The first is that, except at very low irradiance when as expected the 3PGA/triose-P ratio is high in both species, the 3PGA/triose-P ratio is consistently higher in rice than in Arabidopsis (Fig. 4A). This points to a shift in the balance between the light reactions and the CBC to favour the light reactions in Arabidopsis and the CBC in rice. The second is a consistent shift in the relative levels of SBP and FBP, with SBP being higher than FBP in rice and FBP being higher than SBP in Arabidopsis (Fig. 4B). This points to a shift in the balance between FBPase and SBPase, with the former being favoured in rice and the latter in Arabidopsis, and this being compensated by a shift in the levels of their substrates. Alternatively, changes in the relative levels of SBP and FBP might indicate a changed contribution of the newly suggested oxidative pentose phosphate shunt as a source of some of the RuBP (Sharkey and Weise, 2016; Preiser *et al.*, 2019; Sharkey *et al.*, 2020; Xu *et al.*, 2021). The third is that whereas pentose-P and RuBP levels rise progressively with irradiance in Arabidopsis, they plateau at relatively low irradiances in rice (Fig. 4C). In earlier studies with other species, RuBP levels rose progressively in *Phaseolus vulgaris* (bean) leaves (Badger *et al.*, 1984) and the green algae *C. reinhardtii* (Mettler-Altmann *et al*., 2014), whereas RuBP plateaued at irradiances at which photosynthesis was still increasing in wheat (Perchorowicz *et al.*, 1981), *Spinacea oleracea* (spinach) (Dietz and Heber, 1984), and *Raphanus sativus* (radish) (von Caemmerer and Edmondson, 1986). It appears that as irradiance is increased, the rise in the rate of carboxylation is driven in some C_3_ species by increased RuBP regeneration and increased saturation of RuBP-binding sites, presumably accompanied by progressive activation of Rubisco, and in other C_3_ species by removing Rubisco inhibitors and/or by increasing Rubisco activity in other unknown ways.

## Why is there variation in CBC operation between different C_3_ species?

The question arises of why there is so much variation in CBC operation between different C_3_ species. As already mentioned, C_3_ species will have been subject to selective pressure by falling CO_2_ as well as varying temperature and water and nutrient availability. Such pressures combined to drive the evolution of C_4_ photosynthesis in >65 different plant lineages (Sage *et al.*, 2011, 2012; Raven *et al.*, 2017; Sage, 2017). We can be certain (i) that extreme selective pressure was also exerted on the vast majority of terrestrial species that could not enter the evolutionary trajectory toward C_4_ photosynthesis and (ii) that, just as in the evolution of C_4_ photosynthesis, any resulting evolution of the CBC would have occurred in a lineage-dependent manner. Indeed, it is extremely unlikely that only one evolutionary route would have been taken in all C_3_ species. The response in any given species would have been influenced, for example, by (iii) prior conditioning factors both within the CBC and elsewhere, for example, in leaf anatomy; (iv) the light regime, temperature, water availability, and nutrient supply in the ecological niche occupied by the species; and (v) random events. It is also of course possible, and even likely, that some of the current-day variation of CBC operation in C_3_ plants has an even older origin.

CBC metabolite profiling opens up a new research avenue to discover genetic and mechanistic features that underlie interspecies variation in CBC operation, and to understand what specific ecological and evolutionary pressure drove their emergence.

On the one hand, it will be instructive to widen the empirical base by investigating CBC profiles in a wider set of C_3_ species. This would include (i) sets of species that are characteristic of different habitats; (ii) species with short-lived and long-lived leaves and the associated changes in investment strategies (Wright *et al.*, 2004; Donovan *et al.*, 2011); (iii) even more phylogenetically diverse species; and (iv) species that are closely related, or even genotypes from within the same species. The choice of species could be guided by existing knowledge about species- and genotype-dependent variation in photosynthetic traits, both between diverse species (Evans, 1989; Wullschleger, 1993) and in closely related genotypes (Acevedo-Siaca *et al.*, 2020, 2021; McAusland *et al.*, 2020; Silva-Perez *et al.*, 2020). This expanded dataset might allow the most prevalent modes of CBC operation to be defined, and identify which enzymes or sets of enzymes are most frequently affected. It will also be instructive to profile CBC metabolites in a wider range of species that operate different types of CCM including (v) C_4_ species that operate other subtypes in addition to the NADP-ME subtype; (vi) species that represent intermediate stages in the evolution of C_4_ photosynthesis; and (vii) cyanobacteria and eukaryotic algae to learn how operation of their CCMs impacts on CBC function.

In parallel, it will be important to investigate the mechanistic reasons for species differences. These might include investigating expression patterns, protein abundances, and kinetic characteristics of enzymes that act on CBC metabolites or sets of CBC metabolites that show divergent responses between different species, for example, such as those seen between FBPase and SBPase in Arabidopsis and rice (see Fig. 4B and literature cited above), or further metabolic traits and sets of plants that emerge from broader surveys of CBC metabolite profiles. Studies of CBC function in phylogenetically closely related species or genotypes might enable mapping of the underlying genetic variance. It will be very instructive to integrate analyses of CBC metabolite profiles with existing and emerging information about the kinetic and regulatory characteristics of Rubisco (see above) as these might be accompanied by changes in the poising in the remainder of the CBC. A related and potentially very powerful approach would be mining of meta-genome data, which has already been applied to identify functionally important sequence changes between species for Rubisco (see Orr *et al.*, 2016; Davidi *et al.*, 2020). In the future, an analogous approach might be applied to other CBC enzymes.

It will also be important to ask if differences in CBC metabolite profiles are sometimes linked with changes in leaf anatomy or cell ultrastructure. Leaf anatomy varies greatly between species, and is an important determinant of whole-leaf photosynthesis (Niinemets, 2001; Oguchi *et al.*, 2005; Poorter *et al.*, 2009; Terashima *et al.*, 2011; Tsukaya, 2018; Ren *et al.*, 2019). For example, efficient photosynthesis depends on a high chloroplast surface area exposed to intercellular airspace per unit leaf area (Tomás *et al.*, 2013; Théroux-Rancourt and Gilbert, 2017; Ren *et al.*, 2019). One striking difference between the CBC metabolite profiles in the five C_3_ species studied in Arrivault *et al.* (2019) was that CBC metabolite levels were very high on a fresh weight basis in rice and cassava, and that this was linked to a high protein and chlorophyll content per unit fresh weight in these species. High protein content, in particular for proteins in the photosynthesis apparatus, will support a higher rate of photosynthesis and allow more efficient use of light energy, but with the risk of drawing down internal CO_2_ levels and increasing wasteful photorespiration. Rice leaves are characterized by small lobed mesophyll cells with a network of chloroplasts and stromules on the surface that faces the air space, and with mitochondria located in the inner zone of the mesophyll cells (Sage and Sage, 2009; Busch *et al.*, 2013). This special anatomy and ultrastructure results in a high mesophyll transfer conductance and increased efficiency of recapture of photorespired CO_2_. It may prevent internal CO_2_ from being drawn down by the high CBC activity that results from rice high protein and metabolite content per unit leaf mass. It is unclear what anatomical or other features underlie the high protein content in cassava leaves, which has also been noted in other studies (Awoyinka *et al.*, 1995; Nassar and Marques, 2006). Indeed, it may be rewarding to make a more systematic analysis of the CBC and other subprocesses of photosynthesis across species with differing leaf protein content.

## Potential application of CBC metabolite profiling to study adaption to the environment

The studies of Arrivault *et al.* (2019) and Borghi *et al.* (2019) addressed interspecies differences in CBC metabolite profiles, and were made possible because the metabolite profiles were rather robust against short-term changes in conditions such as irradiance. However, it remains possible that other environmental conditions, and especially longer term environmental changes that affect leaf anatomy and composition, may lead to more marked changes in CBC operation within a given species.

Such changes could be detected by profiling CBC metabolites. For example, studies in the 1980s showed that transfer to lower temperature leads to a marked rise in the CBC metabolites that could be measured at that time (Leegood and Furbank, 1986; Sharkey *et al.*, 1986), due at least in part to increased sensitivity of cytosolic FBPase to inhibition by fructose 2,6-bisphosphate (F2,6BP) and AMP at lower temperature (Stitt and Große, 1988). Higher levels of CBC metabolites may partly compensate for the temperature-dependent decrease in catalytic activities of CBC enzymes. Leaves that develop at low temperature have small mesophyll cells with a small vacuole, a high protein content (Huner *et al.*, 1981, 1984), and a 2- to 3-fold higher abundance of all CBC enzymes (Strand *et al.*, 1999), again compensating for the lower catalytic activity of enzymes at low temperature (Stitt and Hurry, 2002). Further environmental conditions such as nitrogen availability (Evans, 1989) and irradiance (Oguchi *et al.*, 2005; Terashma *et al*., 2006, 2011; Théroux-Rancourt and Gilbert, 2017) also result in strong changes of leaf anatomy and composition. Little is known about whether there are accompanying changes in CBC operation.

## Some features of the CBC metabolite profile are conserved across species

**Fig. 5.**
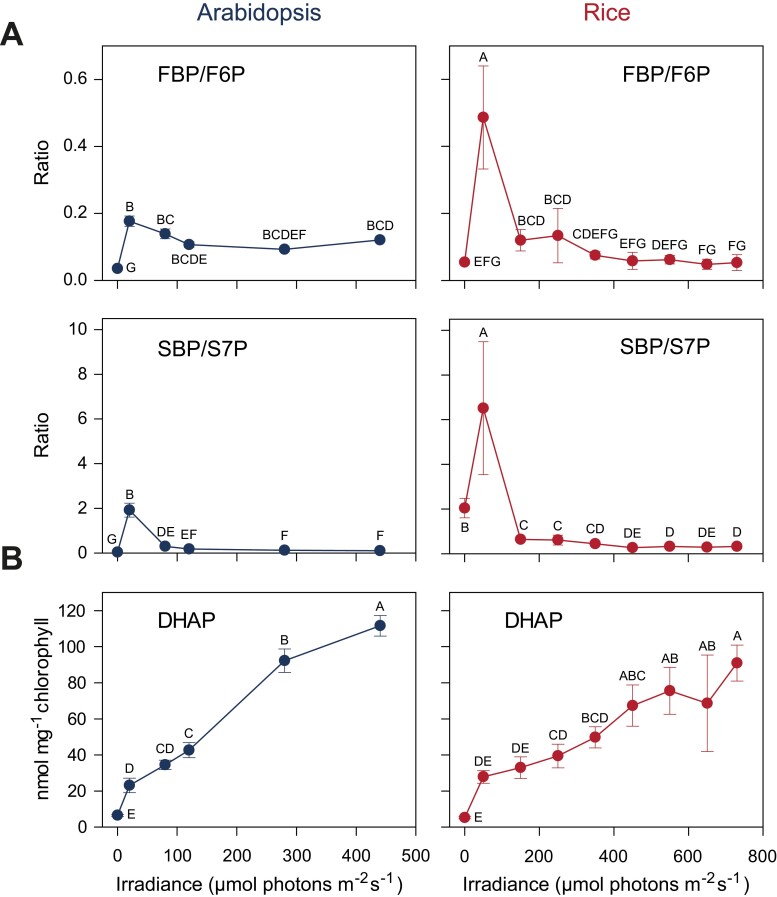
Examples of conserved responses of CBC metabolite levels in Arabidopsis (blue, left-hand panels) and rice (red, right-hand panels). (A) FBP/F6P and SBP/S7P ratios peak at low irradiance and then decline. These are the substrate/product ratios of the two irreversible reactions FBPase and SBPase, respectively. A high substrate/product ratio reveals that the enzyme is restricting flux, even though flux will be very low at these low irradiances close to the compensation point. The implication is that FBPase and SBPase are inhibited, probably because they are not or only weakly activated by post-translational redox activation. (B) DHAP levels are already relatively high at the light compensation point and only rise another 3- to 4-fold at higher irradiance as increasingly fast rates of photosynthesis are achieved. This implies that there is a restriction on the use of triose-P (and other CBC metabolites) for end-product synthesis at very low light. The results are shown as the mean ±SD (*n*=4 in almost all cases). One-way ANOVA with FDR was performed on log-transformed data as in Fig. 4. This was followed by a Tukey’s HSD post-hoc test. The display is modified from Borghi *et al.* (2019). Metabolite abbreviations are as in Fig. 1.

The preceding sections focused on interspecies differences in CBC metabolite profiles and what they tell us about the diversity of the CBC in different C_3_ species. However, the response of the CBC metabolite profile to rising irradiation in Arabidopsis and rice (Borghi *et al.*, 2019) also revealed some shared features. The first conserved feature was that the FBP/F6P and SBP/sedoheptulose 7-phosphate (S7P) ratios peaked at very low irradiance, which was close to the light compensation point, and then declined (Fig. 5A). This points to FBPase and SBPase activity being restricted at low irradiance (see below for further discussion). The second conserved feature was that triose-P rose to relatively high levels at around the light compensation point, but increased only gradually as irradiation rose further and there was a large increase in the rate of photosynthesis (Fig. 5B). Other CBC metabolites were also present in substantial amounts in low light (Borghi *et al.*, 2019). This points to consumption of triose-P for end-product synthesis being strongly restricted in low irradiance.

These two conserved features can also be discerned in earlier studies of CBC metabolite levels. High FBP/F6P and SBP/S7P, and relatively high triose-P were seen at low irradiance in CBC metabolite profiles in *C. reinhardtii* (Mettler-Altmann *et al*., 2014). Relatively high levels of triose-P and other CBC intermediates were also found at the CO_2_ compensation point in Arabidopsis (Arrivault *et al.*, 2009), another condition where there is no net carbon gain in the CBC. With hindsight, high FBP/F6P ratios and relatively high triose-P levels at low irradiance or low CO_2_ (often as generated by applying water or salt stress) can be discerned in earlier more fragmentary analyses of the metabolite levels in wheat, spinach, bean, radish, and sugar beet (Stitt *et al.*, 1980, 1983, 1984; Badger *et al.*, 1984; Dietz and Heber, 1984; von Caemmerer and Edmondson, 1986; Seeman and Sharkey, 1986, 1987; Sharkey and Seeman, 1989; Servaites *et al.*, 1989; compiled in Borghi *et al.*, 2019).

**Fig. 6.**
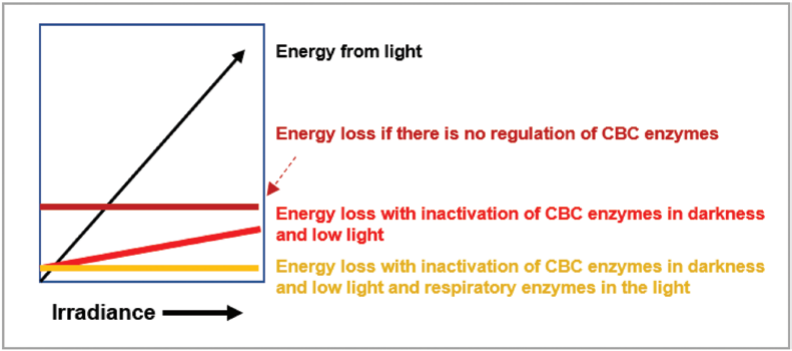
Suppression of futile cycles is especially important in low irradiance. Simultaneous activity of CBC enzymes and respiratory enzymes will lead to futile cycles that would consume ATP and decrease photosynthetic efficiency. To catalyse the high CBC flux that is attained in high light, the activities of CBC enzymes are much higher than those of respiratory enzymes. In the absence of regulation, the rate of futile cycling is therefore likely to be set by the capacity of the respiratory enzymes (schematically illustrated as the dark red line) and in low irradiance may even exceed the energy available from respiration and photosynthetic electron transport (black line). Inactivation of CBC enzymes in darkness and low irradiance (red line) will reduce wasteful futile cycles in low light. Inactivation of respiratory enzymes in the light (yellow line) would further reduce futile cycles in low light and also supress futile cycling in high light when CBC enzymes are activated.

**Fig. 7.**
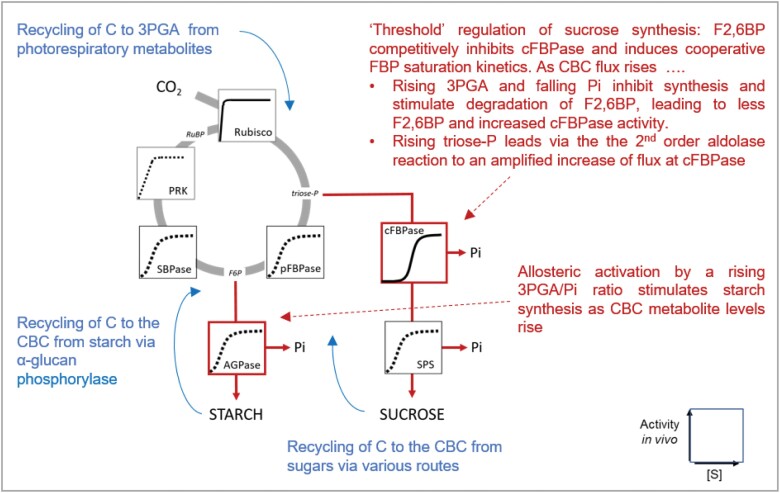
Tight regulation of net carbon maintains CBC metabolites at substantial levels in low light and low CO_2_, allows a rapid increase in net product synthesis as CBC metabolite rise, and maintains levels of metabolites to support CBC flux and energy dissipation via photorespiration in low CO_2_. This is achieved by tight regulation of end-product synthesis (red) and probably by recycling of carbon to the CBC (blue). In steady-state photosynthesis, five out of six of the triose-Ps must be used to regenerate RuBP (actually, more if photorespiration is occurring) and the remainder can be used for end-product synthesis, which recycles Pi and allows continued ATP synthesis. If end-product synthesis is too fast, RuBP will be deleted and CO_2_ fixation will be inhibited. If end-product synthesis is too slow, ATP synthesis will be restricted and CO_2_ fixation inhibited. Sucrose synthesis is regulated by a network including F2,6BP according to a ‘threshold’ principle that inhibits removal of triose-P by cFBPase when triose-P and other CBC metabolites fall below a ‘threshold’ level, and facilitates a steep rise in flux as net CO_2_ fixation rises and CBC metabolites rise above this threshold (see Stitt, 1990 and Stitt *et al.*, 2010 for details). Starch synthesis is stimulated by allosteric activation of AGPase as the CBC metabolite levels rise and Pi falls. At the same time, falling CBC metabolite levels may trigger recycling of carbon from starch and sugars (for details, see text). A short-term shortfall of carbon in the CBC may also be buffered by carbon returning to 3PGA from the large pools of photorespiratory metabolites. The sketch of pathways is modified from Stitt *et al.* (2010). Abbreviations: ADP-glucose pyrophosphorylase (AGPase); cytosolic/plastidic fructose-1,6-bisphosphatase (c/pFBPase); fructose 1,6-bisphosphate (FBP); F2,6BP, fructose 2,6-bisphosphate; inorganic phosphate (Pi); phosphoribulokinase (PRK); substrate concentration ([S]), sedoheptulose-1,7-bisphosphatase (SBPase); sucrose-phosphate synthase (SPS). Metabolite abbreviations are as in Fig. 1.

The identification of these conserved features raises several questions. First, how is flux at FBPase and SBPase constrained in low irradiance? Second, how are triose-P and other CBC metabolites maintained at relatively high levels in low irradiance or low CO_2_ when there is little or no net photosynthesis. Third, what is the functional importance of these conserved features. The next two sections explore the underlying biochemical mechanisms and argue that these conserved features may be important for efficient photosynthesis in low irradiance or when the internal CO_2_ concentration is low due to stomatal closure (Figs 6, 7). In a later section, we argue that these conserved features may also be important for photosynthesis in fluctuating conditions.

As background for the following sections, it should be noted that whilst low irradiance and low CO_2_ are similar in allowing little or no net CO_2_ fixation, there are also fundamental differences between these two conditions. In low light, there is little light energy to drive CBC flux and photorespiration and, presumably, a premium on not wasting energy including minimization of futile cycles between enzymes in photosynthetic and respiratory metabolism. In contrast, in low CO_2_, there is an excess of light energy, and CBC flux is required to supply RuBP not only for carboxylation, which will be slow in these conditions, but also for oxygenation. Low internal CO_2_ will result from stomatal closure, for example under conditions of decreased water availability. In these conditions, it is important to maintain flux in the CBC and in photorespiration, because this consumes excess energy from the light reactions and protects against photoinhibition (Osmond, 1981). Indeed, at least in Arabidopsis in moderate light, metabolite levels at compensation point CO_2_ resemble those in ambient CO_2_, supporting rapid flux in the CBC and in photorespiration, and enabling similar rates of thylakoid electron transport to those in ambient CO_2_ (Arrivault *et al.*, 2009). Furthermore, and in contrast to low irradiance, in low CO_2_, futile cycles might be tolerated; indeed, they might even provide an additional way to dissipate energy.

## Conserved features include regulatory responses that increase photosynthetic efficiency at low irradiance by restricting futile cycles

The high FBP/F6P and SBP/S7P ratios found under low irradiance (Fig. 5A) indicate that plastidic FBPase and SBPase are inhibited in this condition. This is important because the activity of these CBC enzymes would result in wasteful futile cycles of respiratory metabolism and impact severely on the efficiency of photosynthesis. The impact will be much more deleterious in low irradiance, when comparatively little energy is available, than in high irradiance when more light energy is available (Fig. 6). After peaking in very low irradiance, the FBP/F6P and SBP/S7P ratios decreased and then remained rather constant or even continued to fall across a wide range of irradiance where the rate of photosynthesis is increasing (Fig. 5A; Borghi *et al.*, 2019). This observation indicates not only that FBPase and SBPase are largely inactive in low irradiance but also that they are progressively activated to promote CBC flux over a broad range of irradiance.

As already mentioned, plastidic FBPase and SBPase are subject to post-translational redox regulation, and are inactivated in the dark and reduced and activated by thioredoxin in the light (Laing *et al.*, 1981; Scheibe, 1991; Buchanan and Balmer, 2005). The light intensity dependence of this process was investigated for FBPase in the 1980s, by exposing leaves to different irradiances, then extracting them quickly, and immediately assaying FBPase activity. These studies revealed that FBPase activity increases progressively as irradiance is increased (Leegood, 1985*a*, *b*; Woodrow *et al.*, 1985; for a more extensive review, see Knuesting and Scheibe, 2018). Enzyme activity measurements provide indirect evidence about post-translational modification. These conclusions have been confirmed by Yoshida *et al.* (2014), who used MS-based methods to quantify the redox states of cysteine in proteins (Lennicke *et al.*, 2016; Zhang *et al.*, 2016). Working with Arabidopsis, Yoshida and colleagues found that the reduction state of the regulatory cysteine residues of FBPase and SBPase increased in a progressive manner as irradiance was increased. Incidentally, a similar progressive activation occurs for other (e.g. NADP-malate dehydrogenase) but not all (e.g. PRK and cfATP synthase) thioredoxin-regulated enzymes.

Taking together metabolite profiling and studies of the activation and reduction state of enzymes, these results show that post-translational activation of CBC enzymes serves to restrict futile cycles and increase photosynthetic efficiency in low irradiance, and to allow a paced increase in CBC flux as the light intensity rises. Incidentally, it was recently reported that Arabidopsis plastidic phosphofructokinase, AtPFK5, is inactivated in the light by thioredoxin-mediated signalling (Hess *et al.*, 2021; Yoshida and Hisabori, 2021), which will further serve to decrease futile cycling (see Fig. 6). The widespread observation that quantum yield in low irradiance light approaches the theoretical maximum also shows that energy wastage is effectively supressed at low irradiance. Nevertheless, there are non-linearities in the response of CO_2_ uptake to light in the low-irradiance range (Kok, 1949; Tcherkez *et al.*, 2017), and low residual levels of futile cycling might contribute to such non-linearities.

## Conserved features reflect regulatory responses that maintain CBC metabolite levels and stabilize photosynthetic performance in low irradiance or low CO_2_

The relatively high levels of triose-P and other CBC metabolites in very low light (Fig. 5B) and in low CO_2_ (Arrivault *et al.*, 2009; see also literature compilation in Borghi *et al.*, 2019) imply that end-product synthesis is very restricted or inhibited when there is little or no net CO_2_ fixation, and that this inhibition is achieved whilst maintaining CBC metabolites at levels that are much higher than those in the dark. As discussed in this section, this response reflects a regulatory network that (i) optimizes CBC function in different light regimes and (ii) enables energy dissipation in a cycle involving the CBC, the oxygenase reaction of Rubisco, and photorespiration when stomata close and the CO_2_ concentration decreases inside the leaf.

The mechanisms that regulate removal of carbon from the CBC are summarized in Fig. 7. One key aspect is a tight regulation of end-product synthesis. In any given steady-state condition, the rate of withdrawal of metabolites from the CBC must be balanced with the rate of CO_2_ fixation to (i) ensure that enough RuBP is regenerated to allow continued CO_2_ fixation, whilst (ii) avoiding overaccumulation of phosphorylated intermediates, depletion of Pi, and inhibition of ATP synthase (Fig. 5B; see also Edwards and Walker, 1983; Stitt *et al.*, 2010). A regulatory network around the cytosolic FBPase involving F2,6BP strongly restricts removal of triose-P from the CBC until a ‘threshold’ concentration of triose-P is reached, and facilitates a large increase in flux to sucrose when this threshold is exceeded (Figs 5B, 7; see also Herzog *et al.*, 1984; Stitt, 1990; Stitt *et al.*, 2010). This regulatory network (i) ensures that substantial levels of metabolites are maintained in the CBC in the absence of net CO_2_ fixation and (ii) drives a strong increase in flux to sucrose in response to a rising rate of CO_2_ fixation without this requiring a large increase in the levels of CBC metabolites or a concomitant depletion of free stromal Pi. In addition to sucrose synthesis, flux to starch needs to be restricted when net photosynthesis is low and increased when it is high. This is especially so in species where starch is a major product of photosynthesis. Allosteric regulation of ADP glucose pyrophosphorylase by the 3PGA/Pi ratio (Ballicora *et al.*, 2004) will restrict starch synthesis in low irradiance, when most phosphorylated CBC intermediates decline and stromal Pi is high. These concepts were largely developed in *S. oleracea* (spinach) in the 1980s. The conserved response with substantial levels of triose-P and other CBC metabolites in low light or low CO_2_ in many species (see above) is consistent with ‘threshold’ regulation of end-product synthesis representing a general strategy to optimize CBC operation in low light or low CO_2_ across many species.

In addition to ‘threshold’ regulation of end-product synthesis, recent research points to CBC metabolite levels being maintained by several complementary responses, which recycle carbon into the CBC in low-irradiance or low-CO_2_ conditions (Fig. 7). This is discussed in Box 1. Briefly hexose-P may be recycled from starch back into the CBC in low irradiance or low CO_2_, in the latter case via a dedicated pathway for starch degradation involving α-amylase3 and β-amylase1 (Valerio *et al.*, 2011; Seung *et al.*, 2013; Zanella *et al.*, 2016) that are transcriptionally triggered by an increase in abscisic acid (Thalmann *et al.*, 2016). Carbon may also be recycled from sugars, including direct import of hexose-P from the cytosol. Hexose-Ps are not normally transported across the envelope membrane of photosynthetic cells, but in low CO_2_ conditions the glucose 6-phosphate:phosphate translocator (GPT2) is induced in a Redox Responsive Transcription Factor 1 (RRTF1)-dependent manner (Weise *et al.*, 2019). Short-term depletion of CBC metabolite pools may also be buffered by recycling of carbon from pools of photorespiratory metabolites that accumulate to quite high levels during rapid photosynthesis and may provide a carbon reservoir when photosynthesis rates suddenly drop.

**Box 1. Recycling of carbon may also contribute to maintenance of CBC metabolite levels in low irradiance or low CO_2_**

As explained in the main text, one conserved feature of CBC metabolite profiles is the maintenance of relatively high levels of intermediates in low irradiance and even more so in low CO_2_, even though there is little or no net photosynthesis. This response is partly explained by ‘threshold’ regulation of end-product synthesis. In addition, recent research is revealing that recycling of carbon back into the CBC also contributes to maintenance of CBC metabolite levels when net photosynthesis is low. This can occur via recycling of carbon from starch, from sucrose, or other sugars, and also by cabon that has accumulated in the rather large pools of photorespiratory metabolites using the previous hours of photosynthesis (summarized in Fig. 7).

**Recycling of carbon from starch**

Starch typically accumulates in leaves in the light (Stitt and Zeeman, 2010; Smith and Zeeman, 2020). It was widely thought that degradation is inhibited in the light. However, it is now established that there can be substantial starch degradation in the light, with the propensity for degradation rising with time in the light, probably due to circadian regulation (Lu *et al.*, 2005; Weise *et al.*, 2006; Fernandez *et al.*, 2017). A decrease in irradiance leads to a stimulation of starch degradation, especially before dusk (Fernandez *et al.*, 2017). In addition to providing carbon for continued synthesis of sucrose, starch degradation might aid maintenance of CBC metabolite levels. Mobilization of leaf starch usually involves an initial attack on the starch granules by β-amylase3 (BAM3), releasing maltose. This is either exported to the cytosol or, via activity of plastidic disproportionating enzymes, can be converted to a family of α-glucan oligosaccharides in the plastid stroma (Stitt and Zeeman, 2012; Smith and Zeeman, 2020). These glucans could act as a substrate for plastidic α-glucan phosphorylase, which generates glucose 1-phosphate (G1P) which can be fed into the CBC. Another possible substrate for α-glucan phosphorylase might be nascent glucans that have not yet been incorporated into the starch granule, and contain enough carbon to maintain CBC pools during short transients. Whilst it is not known how plastidic α-glucan phosphorylase is regulated, it is plausible that falling CBC metabolite levels and the accompanying increase in Pi may promote the reversible reaction in the direction of G1P formation. Indeed, in experiments with starch-loaded isolated chloroplasts, Stitt and Heldt (1981) showed that there is rapid degradation of starch in the light, and that the contribution of phosphorolysis rises as more Pi is supplied in the medium [high Pi in the medium will drive export of triose-P and, in counterexchange, uptake of Pi via the triose phosphate:phosphate translocator (Heldt *et al.*, 2005), leading to a decrease in metabolite levels and increase of Pi in the stroma].

Starch degradation probably also provides a source of carbon in low CO_2_. Weise et al. (2006, 2011) observed that after a transition to low CO_2_, there is a rise in G1P and glucose 6-phosphate (G6P) in wild-type plants, whereas mutants that are deficient in plastidic α-glucan phosphorylase do not show this increase in G1P and G6P, and also have lower levels of CBC metabolites than wild-type plants in low CO_2_. As in low irradiance, possible substrates for plastidic α-glucan phosphorylase might be nascent glucans that have not yet been incorporated into the starch granule and, later in the day, glucans formed as starch degradation speeds up. However, there is also strong genetic evidence for a dedicated pathway for starch degradation in the light under water stress, which is the condition under which the concentration of CO_2_ in the leaf will fall in the field. It involves an initial attack on the starch granule by α-amylase3 and β-amylase1 (Valerio *et al.*, 2011; Seung *et al.*, 2013; Zanella *et al.*, 2016; Thalmann *et al.*, 2016), and is transcriptionally triggered by an increase in abscisic acid (Thalmann *et al.*, 2016). This water stress-induced pathway has been previously discussed mainly in the context of providing carbon to synthesize sucrose, proline, and further protective osmolytes, but it is possible that it also provides substrates for plastidic α-glucan phosphorylase to support maintenance of CBC metabolite pools. It can be envisaged that abscisic acid may exert a concerted action to (i) decrease stomatal aperture, leading to a lower CO_2_ concentration in the leaf; and (ii) increase starch mobilization, maintain CBC metabolite levels, and support CBC operation in the mesophyll cells, allowing continued photorespiration and energy dissipation.

**Recycling of carbon from sugars**

It is also possible that carbon is sometimes recycled to the CBC from sucrose or reducing sugars in the cytosol or vacuole (Sharkey and Weise, 2016; Preiser *et al.*, 2019; Sharkey *et al.*, 2020, Xu *et al.*, 2021). Recycling of carbon from sugars has often been discussed as a means to balance allocation between sucrose synthesis for export and accumulation of starch as a reserve to, for example, support maintenance and growth in the coming night (Huber, 1989; Kingston-Smith *et al*., 1999; MacRae and Lunn, 2006; Stitt *et al.*, 2010; Mengin *et al.*, 2017). However, it may also allow CBC metabolite pools to be stabilized in conditions when photosynthesis is low. On the one hand, any increase in cytosolic hexose-P that results from remobilization of sugars will lead to an increase of F2,6BP, which will inhibit FBPase and decrease the rate at which triose-Ps are withdrawn from the plastid (Stitt, 1990; Stitt *et al.*, 2010). It is also conceivable that in some situations, rising F2,6BP not only inhibits cytosolic FBPase but also activates pyrophosphate:fructose 6-phosphate phosphotransferase (Stitt, 1990), allowing conversion of hexose-Ps to triose-Ps, followed by their import into the plastid via the triose phosphate:phosphate translocator. A more direct stabilization of the CBC metabolite pools by hexose-P deriving from cytosolic or vacuolar sugars will require import of hexose-P into the plastid. Under most conditions, there is no transfer of hexose-P between the chloroplast and cytosol in leaves (Heldt *et al.*, 2005). However, Weise *et al.* (2019) recently showed that after a sudden transition to low CO_2_, the plastid envelope membrane glucose 6-phosphate:phosphate translocator (GPT2), is induced in a Redox Responsive Transcription Factor 1 (RRTF1)-dependent manner. This finding points towards a link with oxidative stress, possibly due to overenergization in the light reactions in low CO_2_ conditions. This could be relieved by maintaining CBC pools at a high enough level to allow energy dissipation in a photorespiratory cycle; that is, rapid regeneration of RuBP in the CBC to support oxygenation and the operation of photorespiration with concomitant dissipation of energy (Osmond, 1981; Arrivault *et al.*, 2009). Incidentally, induction of GPT2 in low CO_2_ might also facilitate recycling of carbon from starch back to the CBC via a second route that involves maltose export to the cytosol where it is used by cytosolic disproportionating enzyme 2 to produce complex oligosaccharides, which are in turn converted to G1P by cytosolic α-glucan phosphorylase. This G1P would normally be used as a starting point for sucrose synthesis. Induction of GPT2 opens up the possibility that cytosolic hexose-Ps are also cycled back into the CBC in the chloroplast.

**Recycling of carbon that has previously accumulated in photorespiration intermediates**

Another potential source of carbon to maintain CBC pools during rapid transients to low light or low CO_2_ are the large pools of metabolites in the photorespiration pathway, including glycine, serine, and glycerate. The pools contain substantially more carbon than the combined CBC metabolites and turn over with half-lives of the order of 10–15 min (Szecowka *et al.*, 2013; Ma *et al.*, 2014; Arrivault *et al.*, 2017), providing a further source of carbon to buffer CBC metabolite pools after a transition to low light or to low CO_2_. Recycling of photorespiration intermediates will require regulation to allow use of ATP by glycerate kinase, and might be more relevant in low CO_2_ conditions than in low irradiance. Eisenhut *et al.* (2017) have highlighted that photorespiration buffers against stress-related transcriptional changes after transitions to low CO_2_. It is likely that this is partly due to buffering of CBC metabolite pools, which will support rapid RuBP regeneration, photorespiration, and dissipation of energy in low CO_2_ conditions (see above).

Thus, a plethora of regulatory responses could contribute towards stabilizing CBC metabolite levels in low light or low CO_2_. Their relative contribution may depend on the species and conditions. For example, whereas ‘threshold’ regulation of end-product synthesis may suffice to maintain a basal level of CBC metabolites in low irradiance, additional mechanisms including recycling of carbon into the CBC (see Box 1) may be important in low CO_2_ where CBC metabolites are held at higher levels than in low irradiance (see Arrivault *et al.*, 2009, and the data meta-analysis in Borghi *et al.*, 2019). These relatively high CBC pools will allow rapid flux to RuBP, and support in not only the carboxylase but also the oxygenase reaction. Importantly, the latter produces 2PG that is metabolized via photorespiration, with associated dissipation of energy (Osmond, 1981). Indeed, similar rates of electron transport were measured at ambient and compensation point CO_2_ (Arrivault *et al.*, 2009), underlining how efficiently energy can be dissipated by this photorespiration. Recycling of carbon to the CBC may be especially important when CO_2_ concentrations inside the leaf fall so far that the rate of oxygenation exceeds the rate of carboxylation. This will result in a net drain of carbon from the CBC metabolite pools in precisely those conditions where CBC flux must be maintained to support energy dissipation in the photorespiratory cycle. It might be speculated that RRTF1-dependent induction of GPT2 (Weise *et al.*, 2019; see Box 1) could be a response to oxidative pressure in the chloroplast, and serve to facilitate import of carbon from the cytosol, restore CBC metabolite pools, and increase energy dissipation in the photorespiratory cycle. Climate change is leading to hotter and often drier climates, making it pressing to gain a better understanding of features that improve CBC performance when stomata are closed.

## Operation of the CBC in fluctuating conditions such as those found in the field

There is mounting interest in the impact of fluctuating irradiance on photosynthetic efficiency, as this will affect photosynthetic performance in the field. This includes not only changes in sun elevation and cloud cover, but also rapid fluctuations of irradiance in canopies due to flickering of leaves in the wind (Townsend *et al.*, 2018). The speed with which photosynthesis responds to sudden changes in irradiance is thought to be an important factor for photosynthetic efficiency, especially in the dense canopies used in modern agriculture (Tayler and Long, 2017; Vialet-Chabrand *et al.*, 2017; Townsend *et al.*, 2018; Burgess *et al.*, 2019; Tanaka *et al.*, 2019; Wu *et al.*, 2019). Recent research has shown that there is genotype diversity in the speed of response of photosynthesis to a change in the light intensity, and this variation is independent of the genetic variation in steady-state photosynthesis rate (Soleh *et al.*, 2016, 2017; Acevedo-Siaca *et al.*, 2020, 2021; De Souza *et al.*, 2020). Several contributing factors have been investigated in the last years, including loss of cumulative photosynthetic performance due to slow adjustment of energy dissipation mechanisms (Kromdijk *et al.*, 2016; Taylor and Long, 2017, Tanaka *et al.*, 2019; Wu *et al.*, 2019) or slow adjustment of stomatal conductance (Lawson *et al.*, 2012; Vialet-Chabrand *et al.*, 2017; Deans *et al.*, 2019). The cross-species analysis of CBC metabolite profiles in Arrivault *et al.* (2019) and Borghi *et al.* (2019) reveals conserved features that may be linked to regulatory responses that improve CBC performance in fluctuating irradiance.

It has been known since the last century that terrestrial C_3_ species differ in how the CBC responds under fluctuating irradiance (Pearcy, 1990; Pearcy *et al*., 1996; Mott and Woodrow, 2000). Extreme adaptations are found in wild species that are adapted to the understorey of woods and forests, where short-lived sun flecks provide a large part of the total intercepted irradiance. In these plants, the CBC is poised to maintain a very large pool of 3PGA in low light (Pearcy *et al.*, 1994, 2005). This allows full use of the NADPH and ATP that is produced during a short sun fleck without need for turnover of the CBC, which would involve a time lag due to the need to build up CBC intermediate levels and post-translationally activate CBC enzymes.

Responses to light transients have also been investigated in species such as spinach, wheat, and Arabidopsis that live in habitats with less extreme temporal fluctuations in irradiance. After a switch from darkness or low light to high light, it can typically take the order of 1–2 min or longer to establish high rates of photosynthesis (Woodrow and Walker, 1980; Laing *et al.*, 1981; Wirtz *et al.*, 1982; Woodrow *et al.*, 1983, 1985; Sage *et al.*, 1987; Mott and Woodrow, 2000). During this time, CBC metabolite levels rise and enzymes are post-translationally activated. In the case of Rubisco, the rate of activation depends on the abundance of Rubisco activase (Woodrow *et al.*, 1996; Mott *et al.*, 1997; Hammond *et al.*, 1998). As Rubisco activase is present at relatively high abundance, there is trade-off between increasing Rubisco activase abundance to increase the rate of activation of Rubisco protein and increasing the abundance of Rubisco protein (Woodrow and Mott, 1989; Yamori *et al.*, 2012; Carmo-Silva and Salvucci, 2013; Kaiser *et al.*, 2016). Less is known about what determines the speed of activation of other CBC enzymes but, in some cases such as plastidic FBPase and SBPase, it will depend on the levels of their substrates, which promote the post-translational activation by thioredoxin (see Woodrow and Walker, 1980; Laing *et al.*, 1981; Woodrow *et al.*, 1985; Faske *et al.*, 1995; Scheibe, 1991; Stitt *et al.*, 2010; Michelet *et al.*, 2013; Knuesting and Scheibe, 2018). Overall, little is known about the ability of crop plants to rapidly increase CBC flux and fully exploit short periods of high light, whether the speed of the response varies between species or cultivars, and whether other events in the CBC may impact on photosynthetic efficiency in fluctuating light regimes.

## Conserved features in CBC operation reveal regulatory responses that improve photosynthetic efficiency in fluctuating irradiance

**Fig. 8.**
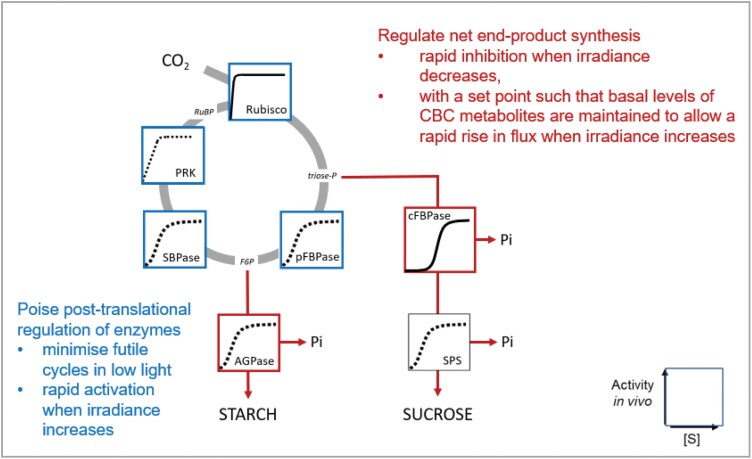
Strategies to maximize the efficiency of CBC operation in fluctuating irradiance. Important features of regulation include (i) effective post-translational and other regulation of CBC enzymes to minimize futile cycles in low irradiance but allow rapid activation of these enzymes to support rapid CBC flux when irradiance increases (blue; for more details, see text and Fig. 6); and (ii) effective inhibition of net end-product synthesis to maintain CBC metabolite levels at a basal level that allows quick re-establishment of CBC flux when irradiance increases (red; for mechanistic details, see text and Fig. 7). Abbreviations are as in Figs 1 and 7.

The preceding sections highlighted two conserved features of CBC operation in low irradiance: a restriction of flux at plastidic FBPase and SBPase due to them being only partially activated; and regulation of end-product synthesis and possibly carbon recycling to maintain a relatively high basal level of triose-P and other CBC metabolites. Both will be important for CBC performance in fluctuating light (Fig. 8).

As already discussed, low post-translational activation of CBC enzymes such as FBPase and SBPase serves to supress futile cycles in low irradiance. This will improve cumulative photosynthetic performance in regimes that are fluctuating between low and high irradiance, and will be especially important when much more time is spent under low irradiance than high irradiance. The amount of futile cycling in the low-irradiance phases will also depend upon how quickly enzymes are inactivated. Inactivation is facilitated by a network including thioredoxins, 2-Cys peroxiredoxins, H_2_O_2_, and NADPH-thioredoxin reductase C (Pérez-Ruiz *et al.*, 2017; Vaseghi *et al.*, 2018; Yoshida and Hisabori, 2018) and can take several minutes, being typically slower than the speed of activation (see, for example, Laing *et al.*, 1981; Wirtz *et al.*, 1982). On the other hand, some enzymes such as PRK are already strongly activated in low light (see above; Yoshida *et al.*, 2014). In this case, activity may be restricted by allosteric regulation by metabolites such as 3PGA, ADP, and RuBP (Gardemann *et al.*, 1983).

Maintenance of a relatively high basal level of CBC metabolites in low irradiance will, for two interlocked reasons, allow a faster rise in CBC flux after a sudden increase in irradiance. First, less time will be needed to build up CBC metabolite levels to a range that supports high enzyme activity. This will also be aided by rigid regulation of end-product synthesis to prioritize use of fixed carbon to build up CBC metabolite pools immediately after the transient to high irradiance rather than using them for end-product synthesis. Second, in many cases such as FBPase and SBPase, the speed or the extent of thioredoxin-dependent redox activation is promoted by binding of their substrates (see above for references). It is also important that the network around F2,6BP that regulates cytosolic FBPase (Stitt, 1990; Stitt *et al.*, 2010) is able to inhibit consumption of triose-P within 5–15 s of darkening (Stitt *et al*, 1983), implying that this network will be able to rapidly slow down or stop removal of carbon from the CBC when irradiance suddenly falls in natural fluctuating light regimes. This will allow rapid establishment of a new steady state with basal levels of CBC metabolites. It would be energetically wasteful to deplete CBC metabolites to very low levels and then replenish them by remobilizing starch or sugars. On the other hand, carbon held in photorespiratory pools such as glycine, serine, and glycerate might provide a useful and less costly source of carbon to replenish the CBC.

In the future it will be interesting to profile CBC metabolites in wild species that are strongly adapted to extreme fluctuating light regimes, as well as in sets of crop cultivars with differing photosynthetic efficiency in fluctuating light (Soleh *et al.*, 2016, 2017¸ Acevedo-Siaca *et al.*, 2020, 2021; De Souza *et al.*, 2020). As already mentioned, a plethora of regulatory responses could contribute towards stabilizing CBC metabolite levels in conditions where net carbon gain is restricted. It will be important to link changes in CBC metabolite profiles with the various strategies that can be deployed to stabilize CBC metabolic pools in low irradiance, and learn if any of them contributes to variance in aggregate photosynthetic performance in fluctuating light regimes. It will be important to investigate a potential trade-off between inactivating CBC enzymes in low irradiance to minimize futile cycling and energy waste, and maintaining a basal level of activation to allow a rapid rise in CBC flux when light suddenly increases. There may also be a trade-off related to the speed of post-translational redox regulation, with faster post-translational regulation of CBC enzymes being advantageous in strongly fluctuating light regimes where it will allow a faster response to a rise in irradiance and less cycling after a sudden decrease in irradiance, and less advantageous in stable light regimes where it may result in increased consumption of redox groups in a cycle of enzyme activation and reduction. Although the mechanistic basis differs, this would be reminiscent of the trade-off between the abundance of Rubisco and Rubisco activase in different kinds of fluctuating irradiance regimes that was outlined (see above) by Mott and Woodrow (2000). Like energy dissipation and stomatal movement, the regulatory features that are likely to impact CBC performance in fluctuating irradiance will differ from those that affect CBC performance in constant irradiance. Collectively, this may explain why genetic variation in photosynthetic performance in fluctuating light is independent of genetic variation in steady-state photosynthesis rate (Soleh *et al.*, 2016, 2017¸ Acevedo-Siaca *et al.*, 2020, 2021; De Souza *et al.*, 2020).

## Conclusions

Profiling of CBC metabolites has uncovered an unexpected degree of diversity in the operation of the CBC in C_3_ species. In retrospect, this diversity is not surprising. All plant lineages have been subject to strong evolutionary pressure from changing CO_2_ and other environmental factors, and in the lineages that did not evolve C_4_ photosynthesis or crassulacean acid metabolism, these pressures will have impacted directly on a CBC that was not shielded by a CCM. Any evolutionary adaptations of the CBC in C_3_ species will have occurred independently in each plant lineage, and will probably have been influenced by specific factors relating to prior history and environmental niche. One important implication is that the best strategy to improve C_3_ photosynthesis might vary from crop to crop. In the future, it will be instructive to apply the top-down approach of CBC metabolite profiling to a much wider range of photosynthetic life forms. This will provide insights into the adaption of the CBC to various types of CCMs, to different environments, and to different plant life histories. The approach can also be applied to panels of closely related species or cultivars of a single species with a varying rate of photosynthesis to learn whether there are accompanying changes in CBC operation and, if so, whether these contribute to the variance in photosynthetic performance. Profiling of CBC metabolites has not only uncovered diversity, it has also highlighted some conserved features of CBC operation. These are likely to be important for photosynthetic performance in conditions where stomata close and internal CO_2_ concentrations fall, a scenario that will be increasingly frequent with future climate change. These conserved features may also be important for photosynthetic performance in low light, and in the fluctuating light regimes that plants experience in the field. Finally, by pinpointing reactions or processes that are divergent or conserved, CBC metabolite profiling provides a starting point for focused biochemical, molecular, and genomics analyses to identify the underlying changes in protein abundance, protein characteristics, or network wiring.

## Abbreviations

CA: carbonic anhydrase
CBC: Calvin–Benson cycle
CCM: carbon-concentrating mechanism
FBP: fructose 1,6-bisphosphate
F2,6BP: fructose 2,6-bisphosphate
FBPase: fructose-1,6-bisphosphatase
FCC: flux control coefficient
F6P: fructose 6-phosphate
G1P: glucose 1-phosphate
hexose-P: hexose phosphate
NAD-ME: NAD-dependent malic enzyme
NADP-ME: NADP-dependent malic enzyme
PC: principal component
Pi: inorganic phosphate
pentose-P: pentose phosphate
PEP: phospho*enol*pyruvate
PEPC: phospho*enol*pyruvate carboxylase
PEPCK: PEP carboxykinase
2PG: 2-phosphoglycolate
3PGA: 3-phosphoglycerate
PRK: phosphoribulokinase
Ru5P: ribulose 5-phosphate
SBP: sedoheptulose 1,7-bisphosphate
SBPase: sedoheptulose-1,7-bisphosphatase
S7P: sedoheptulose 7-phosphate
triose-P: triose phosphate
